# Supplementary material for: Diversity and Molecular Evolution of Antimicrobial Peptides in Caecilian Amphibians
Source: Toxins (Basel). 2024 Mar 14;16(3):150. doi: 10.3390/toxins16030150 (PMC10975883; doi:10.3390/toxins16030150)
Supplement: Supplementary file 1 [file toxins-16-00150-s001.zip › Captions_of_Supplementary_Figures.pdf]

Diversity and molecular evolution of antimicrobial peptides in caecilian amphibians

Captions of Supplementary Figures

Figure S1. Schematic representation of the three situations that we encountered after the alignment of target sequences (genomic/transcriptomic source data, grey bars) against query data from the APD3 database (AMP, green bars). a) The alignment occurs along the entire length of both sequences, b) The AMP aligns in a single block with part of the target sequence; and c) several AMPs match on different parts of the sequence (this is the case of some histone-H2A-derived AMPs).

Figure S2. ML phylogeny of liver-expressed antimicrobial peptide (LEAP2) candidate AMPs using best-fit Dayhoff+G4 substitution model. None of the internal branches have support values above 80 and 95 for SH-aLRT and UFBoot, respectively. Sequences are referenced to APD3 records.

Figure S3. ML phylogeny of lysozyme-C candidate AMPs using best-fit WAG+I+G4 substitution model. Grey circles represent significant support values above 80 and 95 for SH-aLRT and UFBoot, respectively. Sequences are referenced to APD3 records.

Figure S4. ML phylogeny of cathelicidin candidate AMPs using best-fit JTT+G4 substitution model. Grey circles represent significant support values above 80 and 95 for SH-aLRT and UFBoot, respectively. Sequences are referenced to UniProt records.

Figure S5. ML phylogeny of A1P/Antitrypsin candidate AMPs using best-fit LG+G4 substitution model. Grey circles represent significant support values above 80 and 95 for SH-aLRT and UFBoot, respectively. Sequences are referenced to APD3 records.

Figure S6. ML phylogeny of adrenomedulin candidate AMPs using best-fit JTT+G4 substitution model. Grey circles represent significant support values above 80 and 95 for SH-aLRT and UFBoot, respectively. Sequences are referenced to APD3 records.

Figure S7. ML phylogeny of beta-amyloid candidate AMPs using best-fit Dayhoff+I substitution model. None of the internal branches have support values above 80 and 95 for SH-aLRT and UFBoot, respectively. Sequences are referenced to APD3 records.

Figure S8. ML phylogeny of chemokine derived AMPs (group of sequences more similar to APD3 record AP03058) best-fit JTTDCmut+I+G4 substitution model. Grey circles represent significant support values above 80 and 95 for SH-aLRT and UFBoot, respectively. Sequences are referenced to APD3 records.

Figure S9. ML phylogeny of chemokine derived AMPs (group of sequences more similar to APD3 record AP03176) using best-fit JTT+I+G4 substitution model. Grey circles represent significant support values above 80 and 95 for SH-aLRT and UFBoot, respectively. Sequences are referenced to APD3 records.

Figure S10. ML phylogeny of chrombacin/secretogranin candidate AMPs using best-fit HIVb+G4 substitution model. None of the internal branches have support values above 80 and 95 for SH-aLRT and UFBoot, respectively. Sequences are referenced to APD3 records.

Figure S11. ML phylogeny of enolase candidate AMPs using best-fit WAG+G4 substitution model. Grey circles represent significant support values above 80 and 95 for SH-aLRT and UFBoot, respectively. Sequences are referenced to APD3 records.

Figure S12. ML phylogeny of sequences of GADPH derived candidate AMPs using best-fit cpREV+G4 substitution model. Grey circles represent significant support values above 80 and 95 for SH-aLRT and UFBoot, respectively. Sequences are referenced to APD3 records.

Figure S13. ML phylogeny of granulin candidate AMPs using best-fit LG+G4 substitution model. None of the internal branches have support values above 80 and 95 for SH-aLRT and UFBoot, respectively. Sequences are referenced to APD3 records.

Figure S14. ML phylogeny of histone-H2A candidate AMPs (acipensin 6 region) using best-fit LG+G4 substitution model. Grey circles represent significant support values above 80 and 95 for SH-aLRT and UFBoot, respectively. Sequences are referenced to APD3 records.

Figure S15. ML phylogeny of histone-H2A candidate AMPs (hipposin region) using best-fit JTT+G4 substitution model. Grey circles represent significant support values above 80 and 95 for SH-aLRT and UFBoot, respectively. Sequences are referenced to APD3 records.

Figure S16. ML phylogeny of histone-H2B candidate AMPs using best-fit JTT substitution model. Grey circles represent significant support values above 80 and 95 for SH-aLRT and UFBoot, respectively. Sequences are referenced to APD3 records.

Figure S17. ML phylogeny of histone-H3 candidate AMPs using best-fit JTT substitution model. Grey circles represent significant support values above 80 and 95 for SH-aLRT and UFBoot, respectively. Sequences are referenced to APD3 records.

Figure S18. ML phylogeny of IBP candidate AMPs using best-fit FLU+G4 substitution model. Grey circles represent significant support values above 80 and 95 for SH-aLRT and UFBoot, respectively. Sequences are referenced to APD3 records.

Figure S19. ML phylogeny of neuropeptide-W candidate AMPs using best-fit FLU substitution model. None of the internal branches have support values above 80 and 95 for SH-aLRT and UFBoot, respectively. Sequences are referenced to APD3 records.

Figure S20. ML phylogeny of neuropeptide-YY derived candidate AMPs using best-fit FLU+G4 substitution model. Grey circles represent significant support values above 80 and 95 for SH- aLRT and UFBoot, respectively. Sequences are referenced to APD3 records.

Figure S21. ML phylogeny of PACAP family AMPs using best-fit JTT+G4 substitution model. Grey circles represent significant support values above 80 and 95 for SH-aLRT and UFBoot, respectively. Sequences are referenced to APD3 records.

Figure S22. ML phylogeny of POMC candidate AMPs using best-fit JTTDCMut+G4 substitution model. None of the internal branches have support values above 80 and 95 for SH-aLRT and UFBoot, respectively. Sequences are referenced to APD3 records.

Figure S23. ML phylogeny of proenkephalin candidate AMPs using best-fit JTT+G4 substitution model. None of the internal branches have support values above 80 and 95 for SH-aLRT and UFBoot, respectively. Sequences are referenced to APD3 records.

Figure S24. ML phylogeny of thymosin candidate AMPs using best-fit LG+G4 substitution model. Grey circles represent significant support values above 80 and 95 for SH-aLRT and UFBoot, respectively. Sequences are referenced to APD3 records.

Figure S25. ML phylogeny of vasostatin candidate AMPs using best-fit JTT+G4 substitution model. Grey circles represent significant support values above 80 and 95 for SH-aLRT and UFBoot, respectively. Sequences are referenced to APD3 records.

Figure S26. ML phylogeny of cystatin candidate AMPs using best-fit JTT+G4 substitution model. Grey circles represent significant support values above 80 and 95 for SH-aLRT and UFBoot, respectively. Sequences are referenced to APD3 records.

Figure S27. ML phylogeny of kininogen candidate AMPs using best-fit JTT+G4 substitution model. Grey circles represent significant support values above 80 and 95 for SH-aLRT and UFBoot, respectively. Sequences are referenced to APD3 records.
