# Supplementary material for: Diversity and Molecular Evolution of Antimicrobial Peptides in Caecilian Amphibians
Source: Toxins (Basel). 2024 Mar 14;16(3):150. doi: 10.3390/toxins16030150 (PMC10975883; doi:10.3390/toxins16030150)
Supplement: Supplementary file 1 [file toxins-16-00150-s001.zip › Supplementary_Table_S1.pdf]

Table S1. Summary of curated Blast matches of AMPs with the genomic or transcriptomic data of caecilians (gold), anurans (orange), and lungfish (blue). Only those AMPs that matched with at least one caecilian genome or transcriptome are shown.

			Rhinatrema bivittatum^{G+T}	*Ichthyophis kotahoensis*^{T}	*Geotrypetes seraphini*$^{G+T}$	*Microcaecilia unicolor*$^{G+T}$	*Microcaecilia dermatophaga*^{T}	*Caecilia tentaculata*^{T}	*Typhlonectes compressicauda*^{T}	*Typhlonectes natans*^{T}	*Xenopus laevis*^{G}	*Bombina bombina*^{G}	*Protopterus annectens*^{G}
AP01479	Adrenomedullin	Mammalia	X		X	X	X	X	X		X	X	X
AP02917	Amylin/Calcitonin	Fish			X	X			X		X	X	X
AP02517	A1P/ Antitrypsin	Sauropsida	X	X		X	X		X	X	X	X	X
AP03463	Apelin	Mammalia				X					X		
AP01676	Beta-amyloid	Mammalia	X	X	X	X	X	X	X		X	X	X
K7FKZ8*	Cathelicidin	Reptilia	X								X	X	
K7FPA2*	Cathelicidin	Sauropsida				X							
K7GI21*	Cathelicidin	Sauropsida	X		X	X	X	X	X			X	
K7GIB1*	Cathelicidin	Sauropsida	X			X	X				X	X	
M7BBJ0*	Cathelicidin	Sauropsida	X		X	X	X	X	X		X	X	
AP00612	Chrombacin/ Secretogranin	Mammalia	X		X	X	X	X	X	X	X	X	X
G3VCA4*	Cystatin	Mammalia	X		X	X	X	X	X		X		X
H3A036*	Cystatin	Actinistia	X		X	X	X	X	X		X	X	X
Q5M8F3*	Cystatin	Amphibia	X		X	X	X	X	X		X		
AP03058	Chemokine	Fish	X	X	X	X	X				X		X
AP03176	Chemokine	Fish	X		X	X	X		X			X	X
AP03323	Cofilin	Fish	X	X	X	X	X	X	X	X	X	X	X
AP00208	DBI	Mammalia	X		X	X	X	X	X	X	X	X	X
AP03806	Enolase	Sauropsida	X		X	X	X	X	X	X	X	X	X
AP02012	GADPH	Fish	X	X	X	X	X	X	X	X	X	X	X
AP02680	GADPH	Fish				X			X				X
AP00294	Granulin	Mammalia	X		X	X	X	X	X	X	X	X	X
AP00307	Histone H2A	Amphibia	X	X		X	X	X	X	X			
AP00489	Histone H2A	Fish	X	X	X	X					X	X	X
AP02811	Histone H2A	Fish							X		X		X
AP02813	Histone H2A	Fish					X	X	X	X		X	
AP02808	Histone H2B	Mammalia	X		X	X	X	X	X		X	X	X
AP02809	Histone H3	Mammalia	X	X	X	X	X	X	X		X	X	X
AP03415	IBP	Mammalia	X	X	X	X	X	X	X	X	X	X	X

G1PML8*	Kininogen	Mammalia	X		X	X	X	X	X		X	X	X
AP01646	LEAP2	Fish	X	X	X	X					X		
AP03119	LEAP2	Fish		X		X							
AP03568	LEAP2	Fish						X					X
AP02257	Lysozyme C	Mammalia	X		X	X	X	X	X	X		X	
AP02766	Lysozyme C	Mamma	X		X	X		X	X		X	X	
AP03456	Lysozyme C	Sauropsida	X		X	X	X	X	X	X	X	X	
AP02743	Lysozyme G	Fish	X		X	X	X	X	X		X	X	X
AP03464	Neuropeptide W	Mammalia	X			X			X		X		X
AP00400	Neuropetide YY	Amphibia	X	X	X	X		X	X		X	X	X
AP03279	PACAP	Fish	X		X	X	X	X	X		X	X	X
AP03465	POMC	Mammalia	X		X	X					X	X	X
AP00812	Proenkephalin	Mammalia	X			X		X	X		X	X	
AP02896	Thymosin	Fish	X		X	X					X	X	X
AP02353	Vasostatin	Mammalia	X	X	X	X	X	X	X		X		X

[G] species with genome data only
[G+T] species with genomic and transcriptomic data
[T] species with transcriptomic data only
* Blast matches against UniProt records (all other without asterisk are matches against APD3 records)
