# Supplementary material for: Diversity and Molecular Evolution of Antimicrobial Peptides in Caecilian Amphibians
Source: Toxins (Basel). 2024 Mar 14;16(3):150. doi: 10.3390/toxins16030150 (PMC10975883; doi:10.3390/toxins16030150)
Supplement: Supplementary file 1 [file toxins-16-00150-s001.zip › Supplementary_Table_S2.pdf]

Table S2. Reliability of AI-based activity prediction methods used to measure the antimicrobial properties of a dataset of 1000 randomly generated peptides. The percentage of positive hits among the randomly generated data (identifying a sequence as AMP when it is not) is shown for each of the tools.

Server	DBAASP	Antimicrobial Peptide Scanner		CAMPR4		
Method	DBAASP	RF	Earth	RF	SVM	ANN
% of positive hits among the randomly generated data	6.72	6.6	14.4	18.4	8.2	7.2
