# Supplementary figures and images for: Diversity and Molecular Evolution of Antimicrobial Peptides in Caecilian Amphibians
Source: Toxins (Basel). 2024 Mar 14;16(3):150. doi: 10.3390/toxins16030150 (PMC10975883; doi:10.3390/toxins16030150)

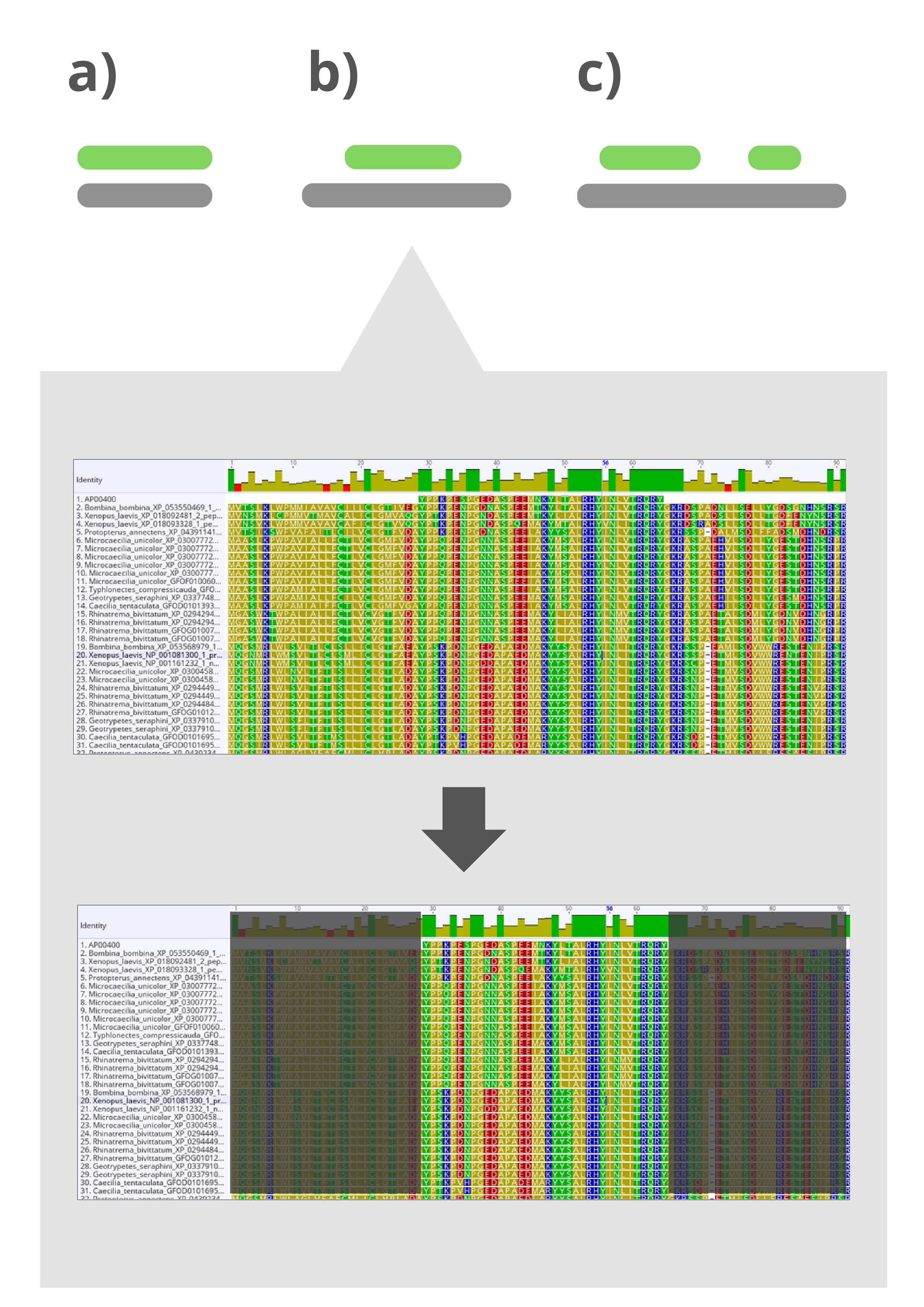

Supplement: Supplementary file 1 [file toxins-16-00150-s001.zip › Supplementary_Figure_S1.jpg]

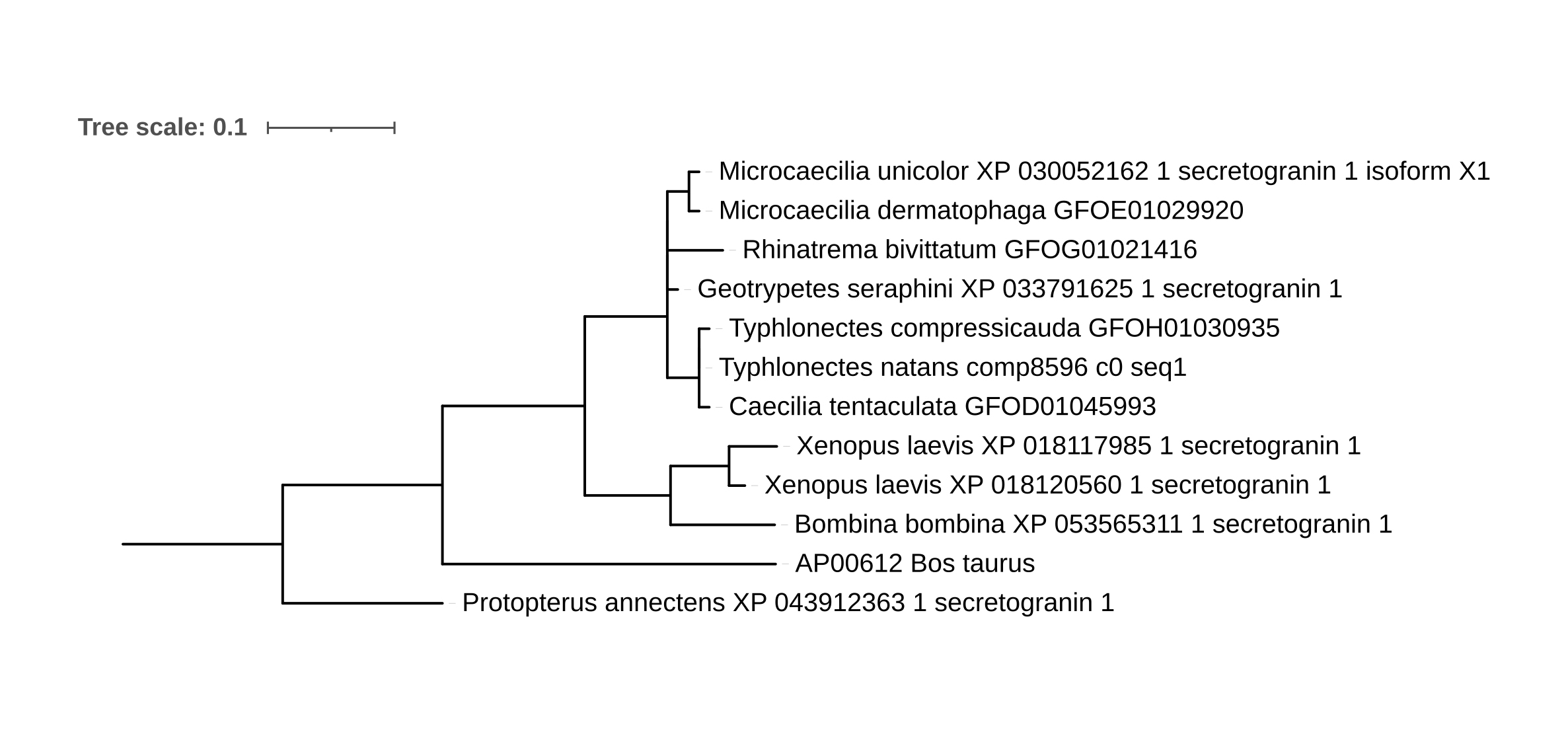

Supplement: Supplementary file 1 [file toxins-16-00150-s001.zip › Supplementary_Figure_S10.jpg]

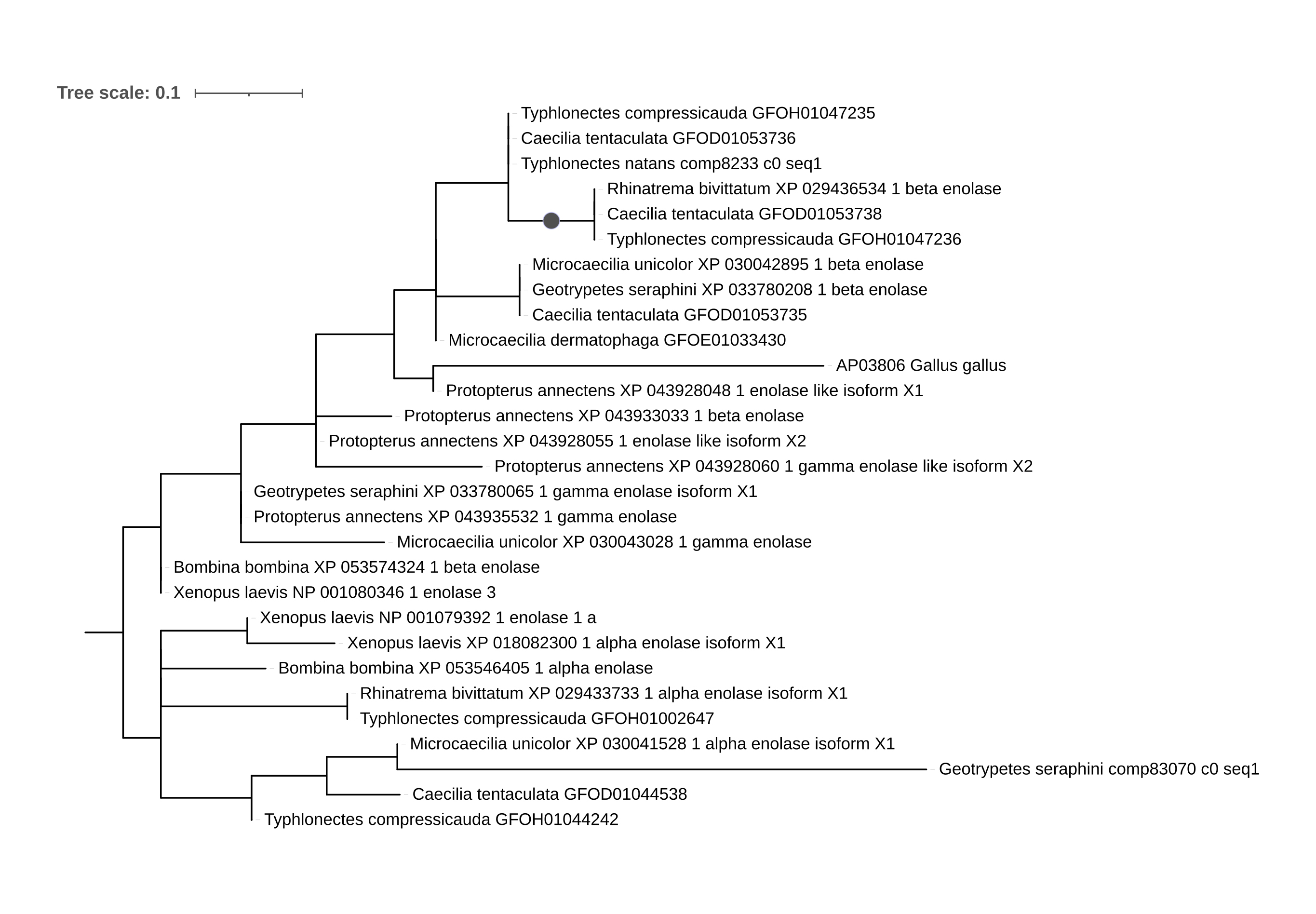

Supplement: Supplementary file 1 [file toxins-16-00150-s001.zip › Supplementary_Figure_S11.jpg]

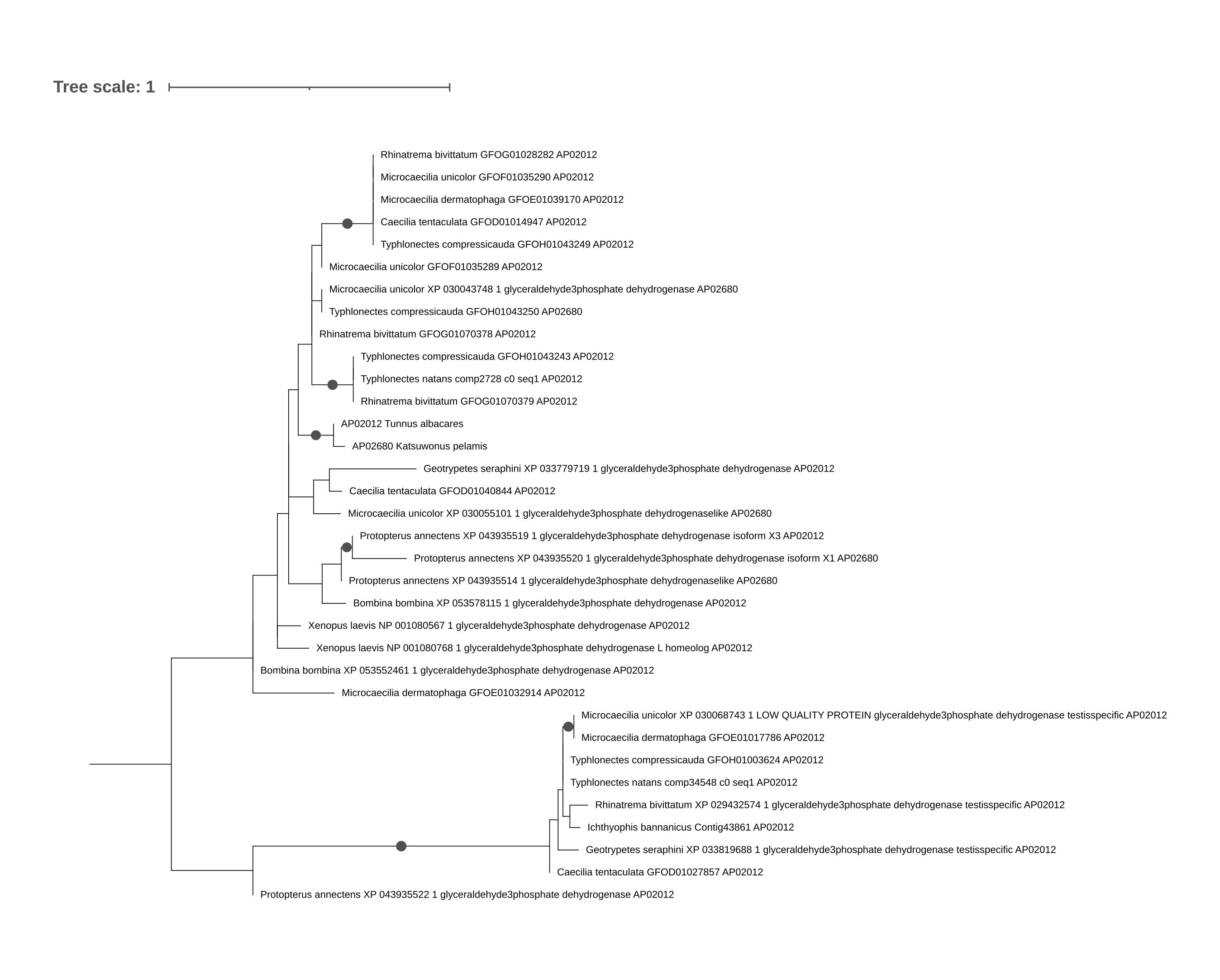

Supplement: Supplementary file 1 [file toxins-16-00150-s001.zip › Supplementary_Figure_S12.jpg]

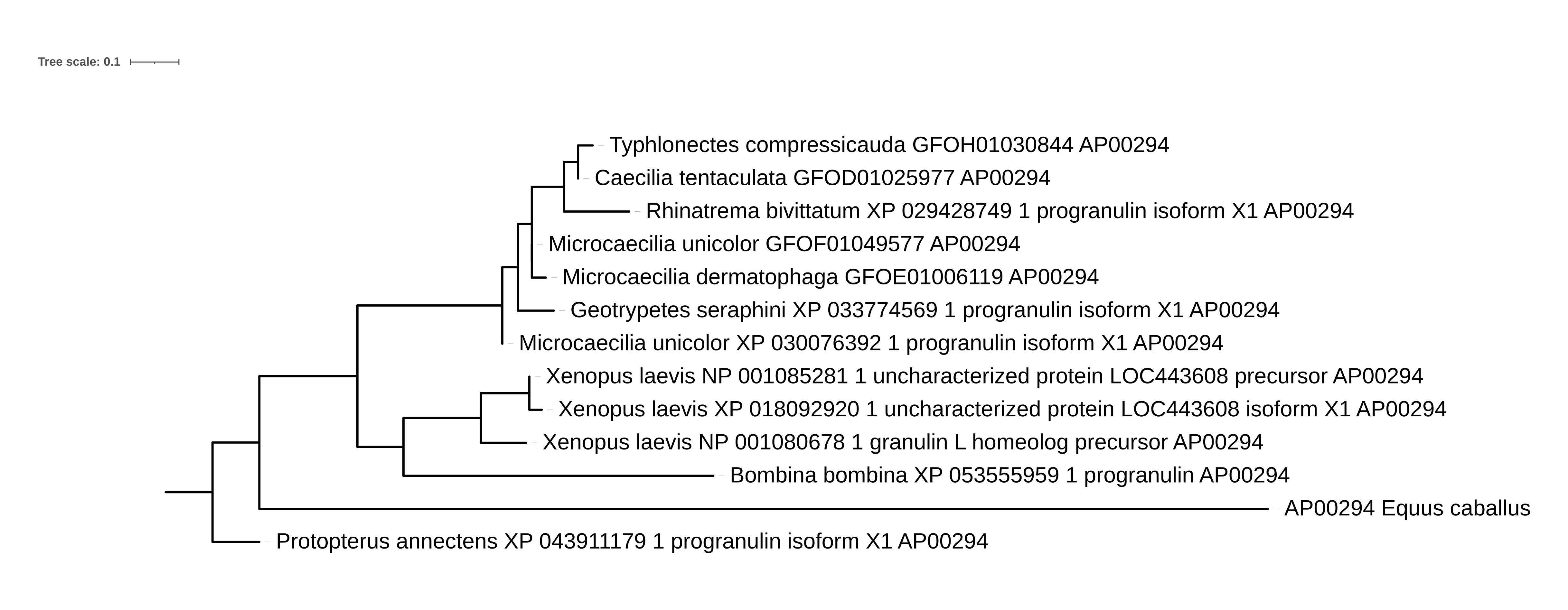

Supplement: Supplementary file 1 [file toxins-16-00150-s001.zip › Supplementary_Figure_S13.jpg]

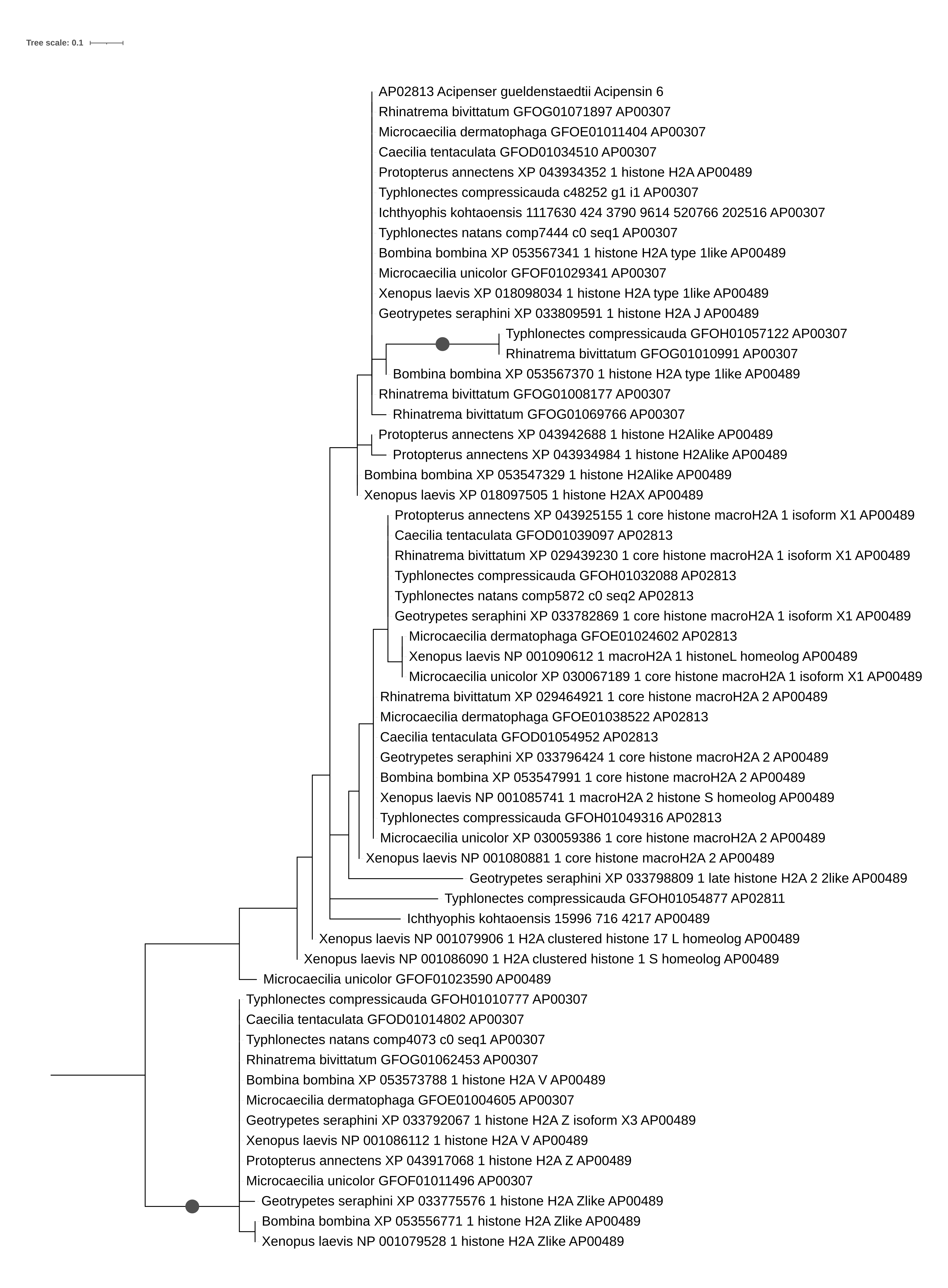

Supplement: Supplementary file 1 [file toxins-16-00150-s001.zip › Supplementary_Figure_S14.jpg]

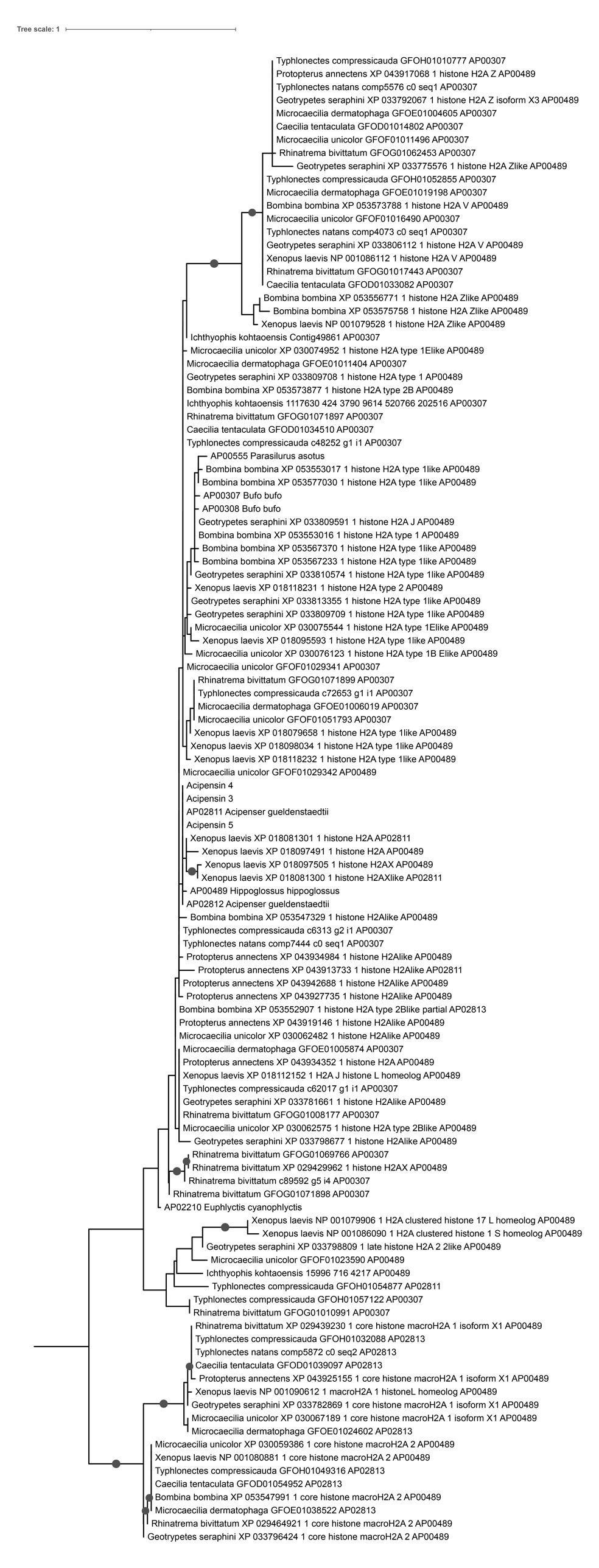

Supplement: Supplementary file 1 [file toxins-16-00150-s001.zip › Supplementary_Figure_S15.jpg]

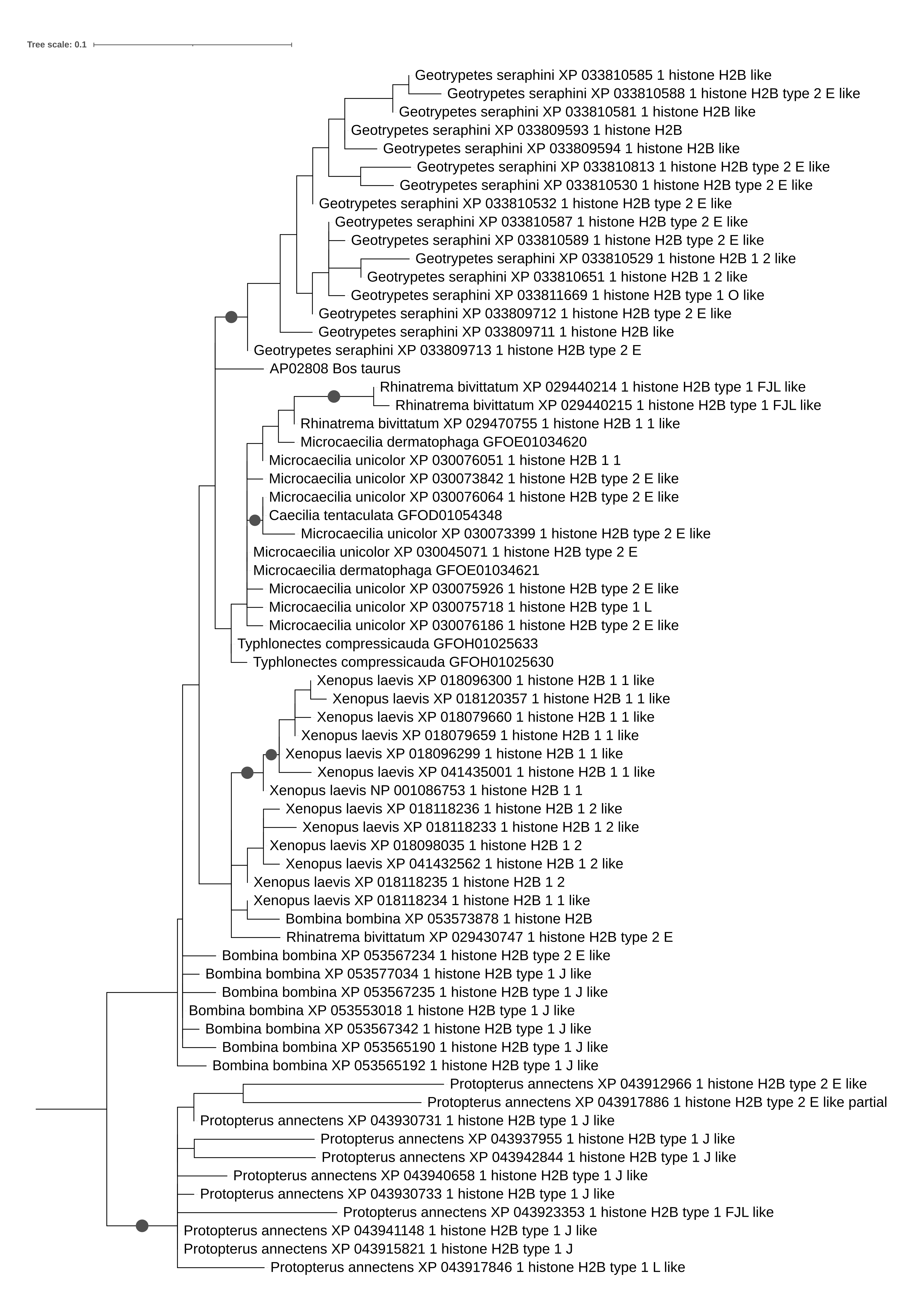

Supplement: Supplementary file 1 [file toxins-16-00150-s001.zip › Supplementary_Figure_S16.jpg]

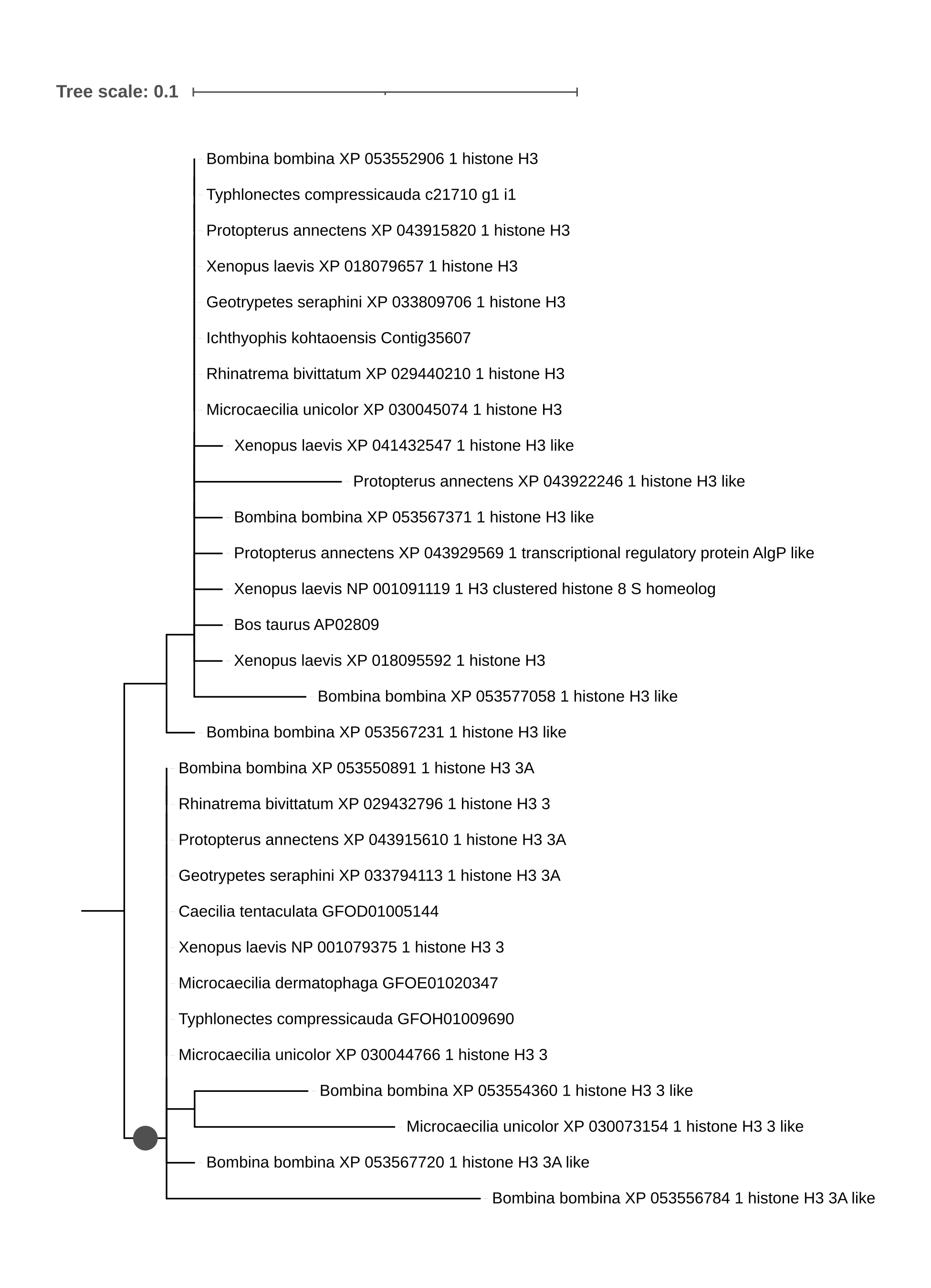

Supplement: Supplementary file 1 [file toxins-16-00150-s001.zip › Supplementary_Figure_S17.jpg]

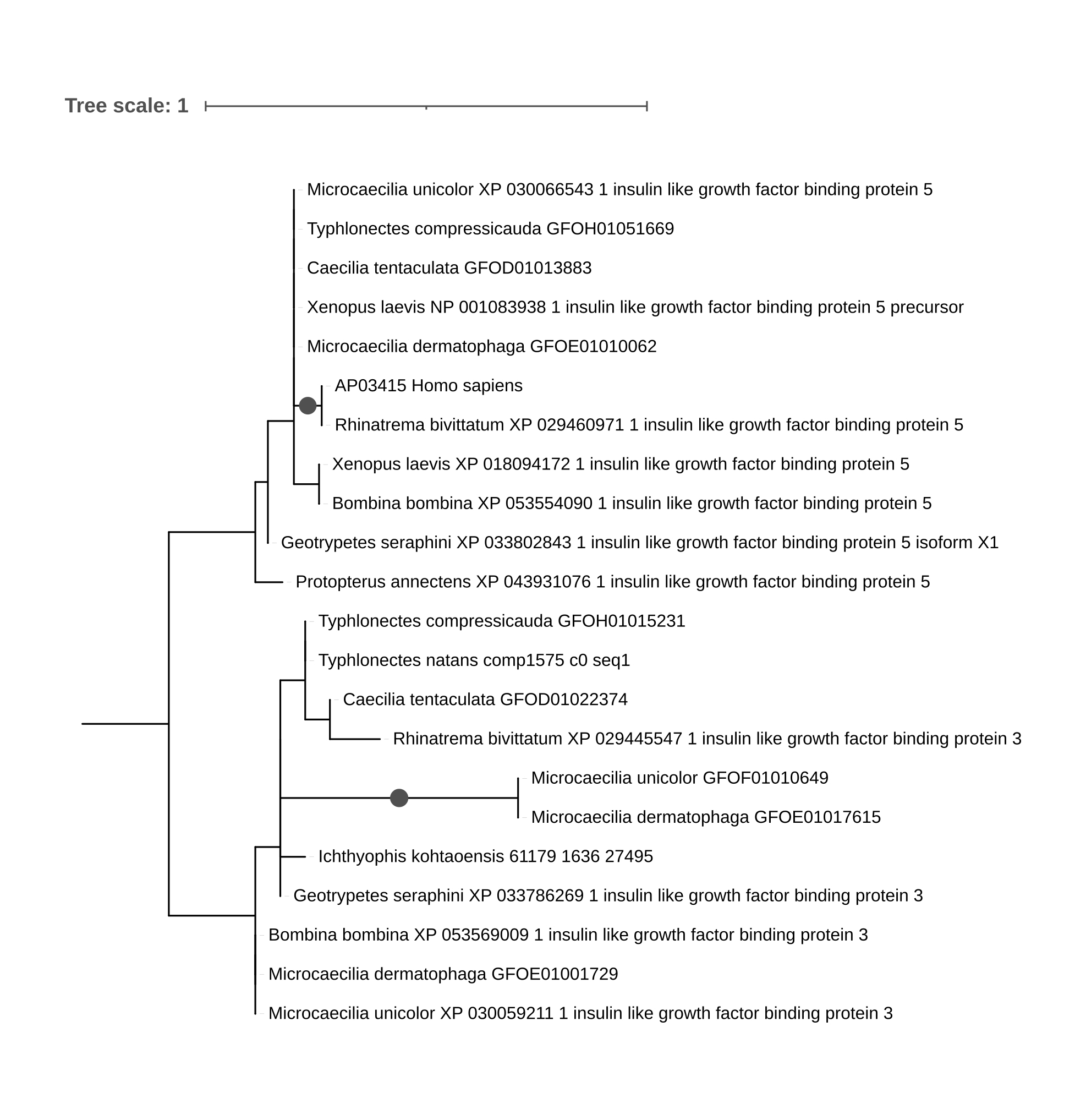

Supplement: Supplementary file 1 [file toxins-16-00150-s001.zip › Supplementary_Figure_S18.jpg]

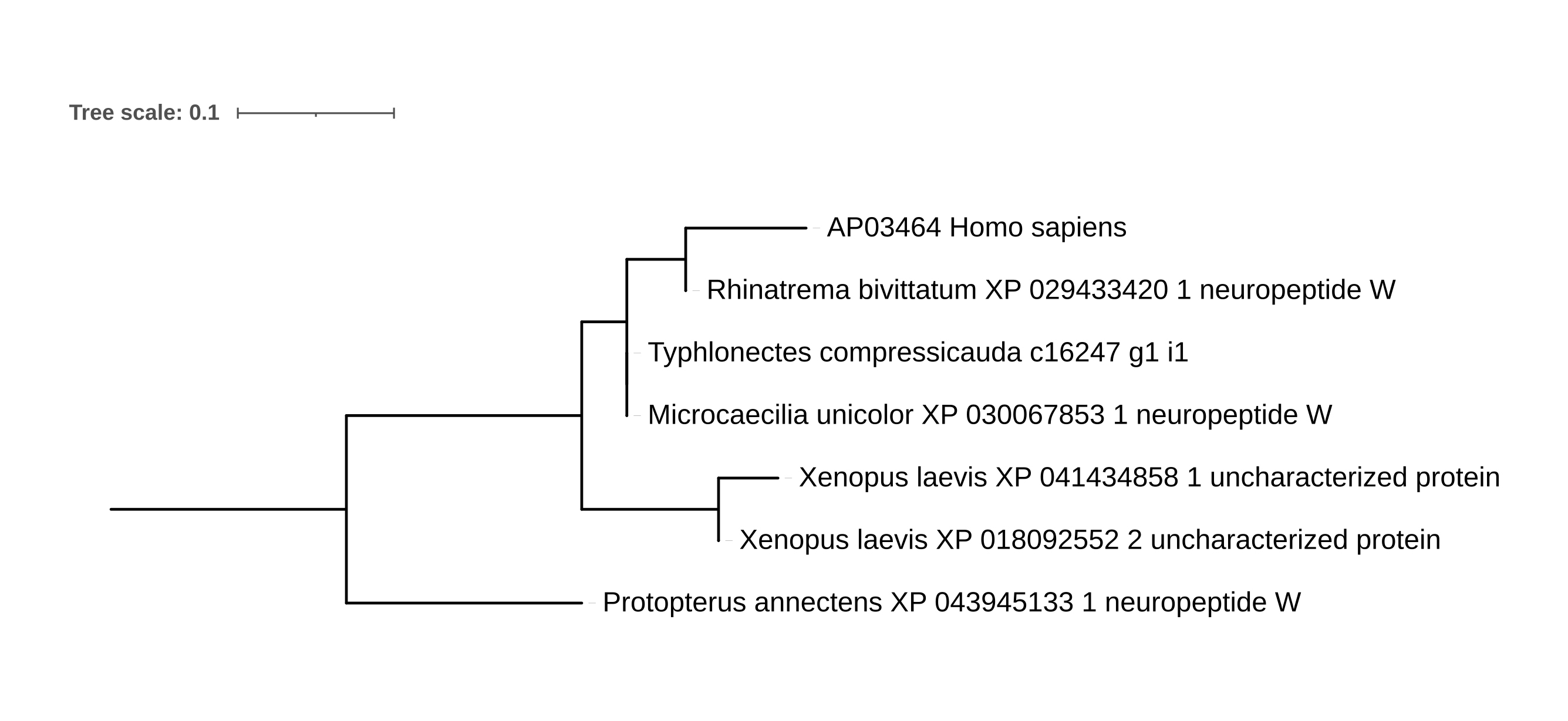

Supplement: Supplementary file 1 [file toxins-16-00150-s001.zip › Supplementary_Figure_S19.jpg]

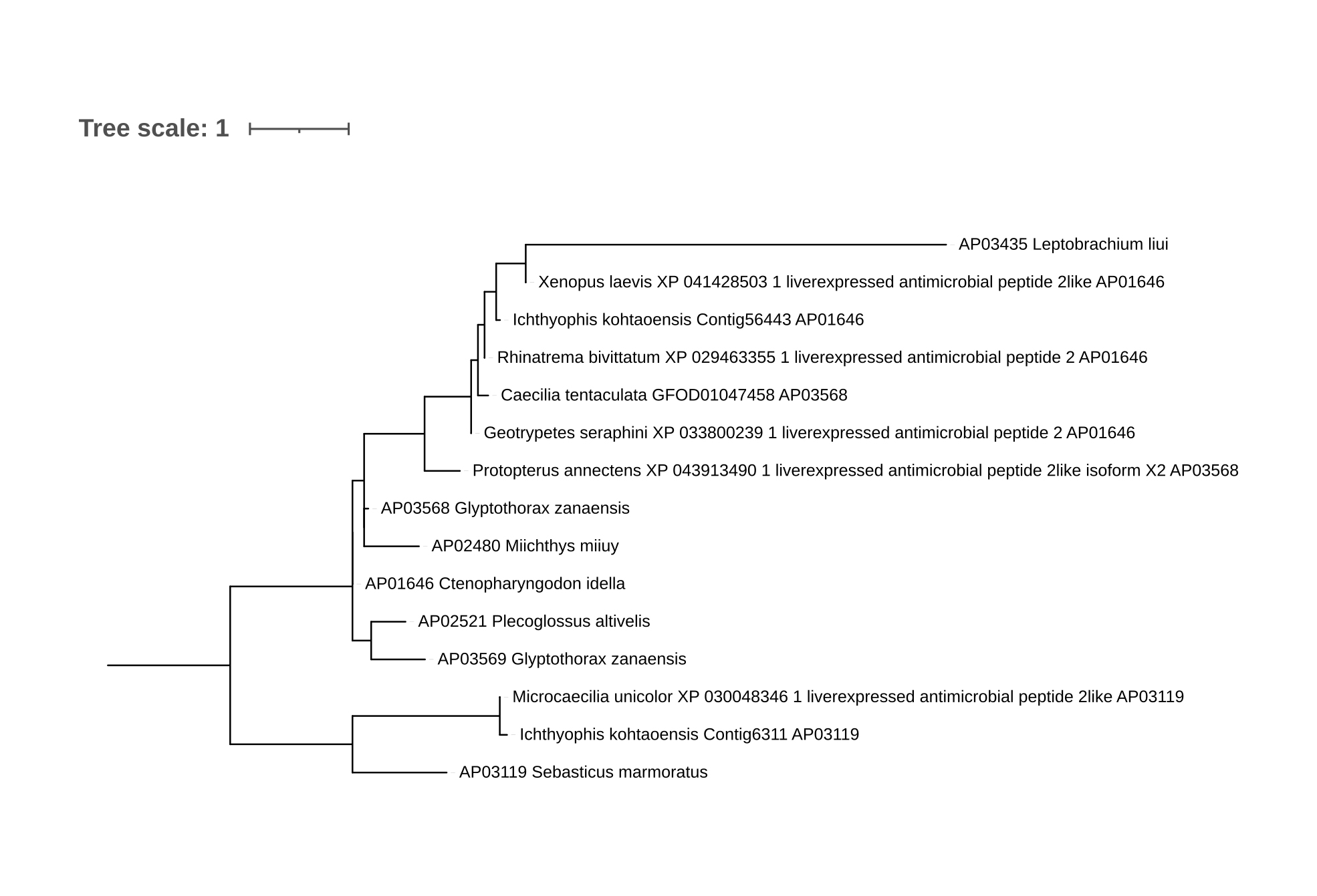

Supplement: Supplementary file 1 [file toxins-16-00150-s001.zip › Supplementary_Figure_S2.jpg]

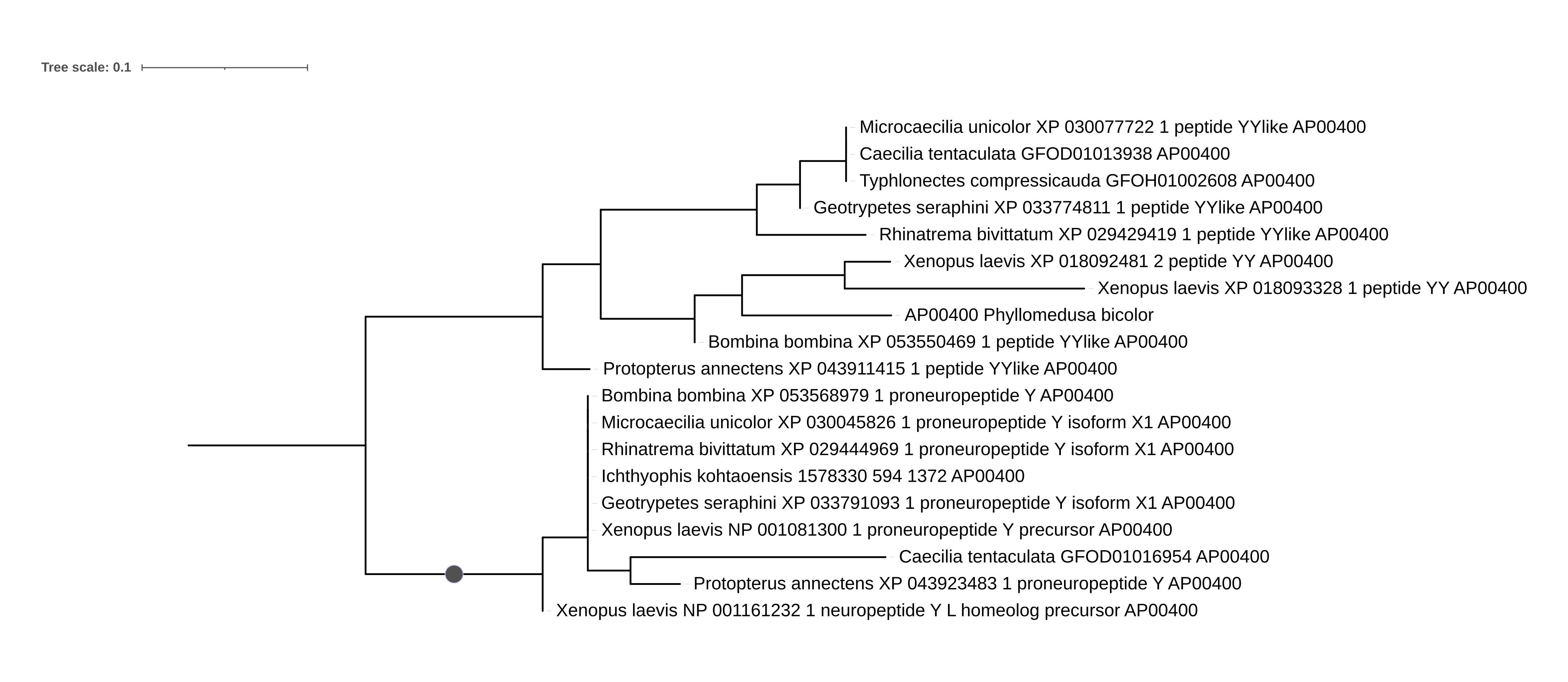

Supplement: Supplementary file 1 [file toxins-16-00150-s001.zip › Supplementary_Figure_S20.jpg]

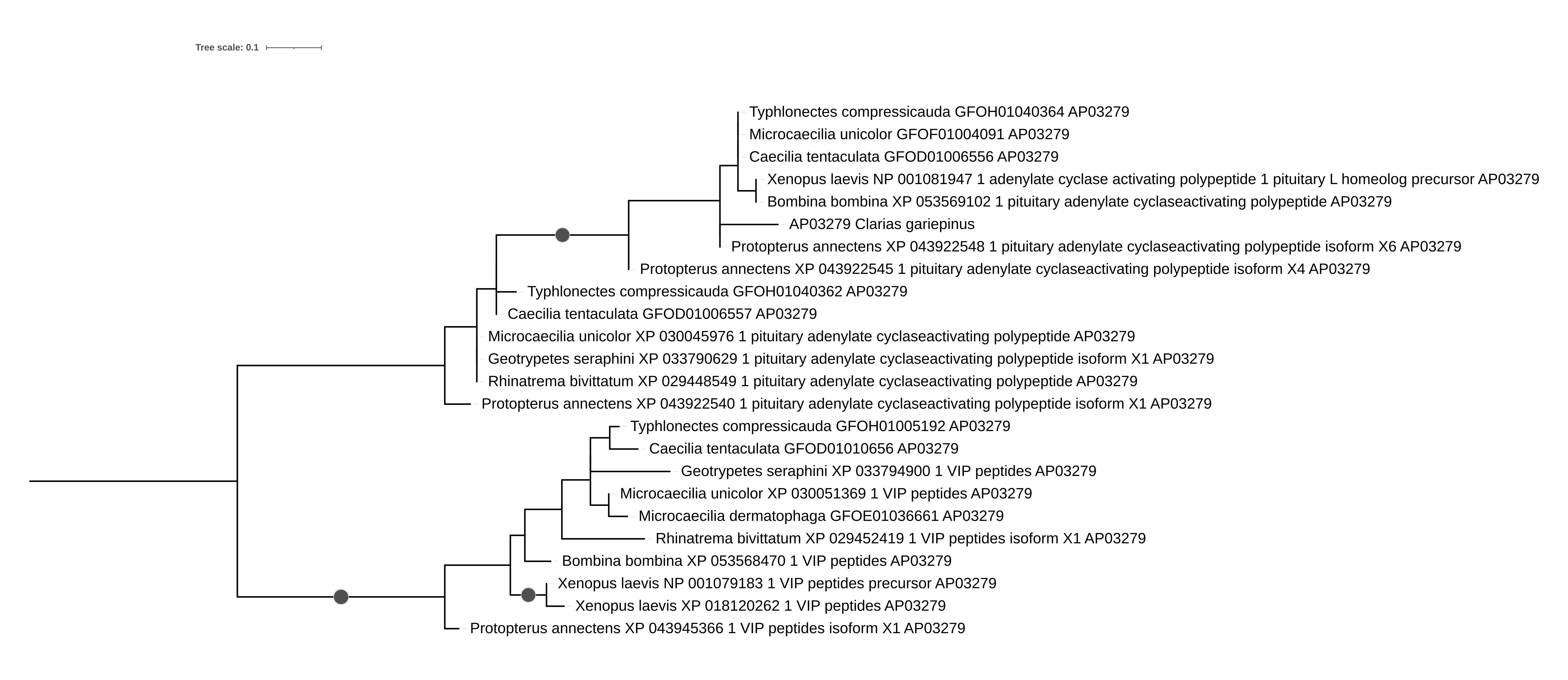

Supplement: Supplementary file 1 [file toxins-16-00150-s001.zip › Supplementary_Figure_S21.jpg]

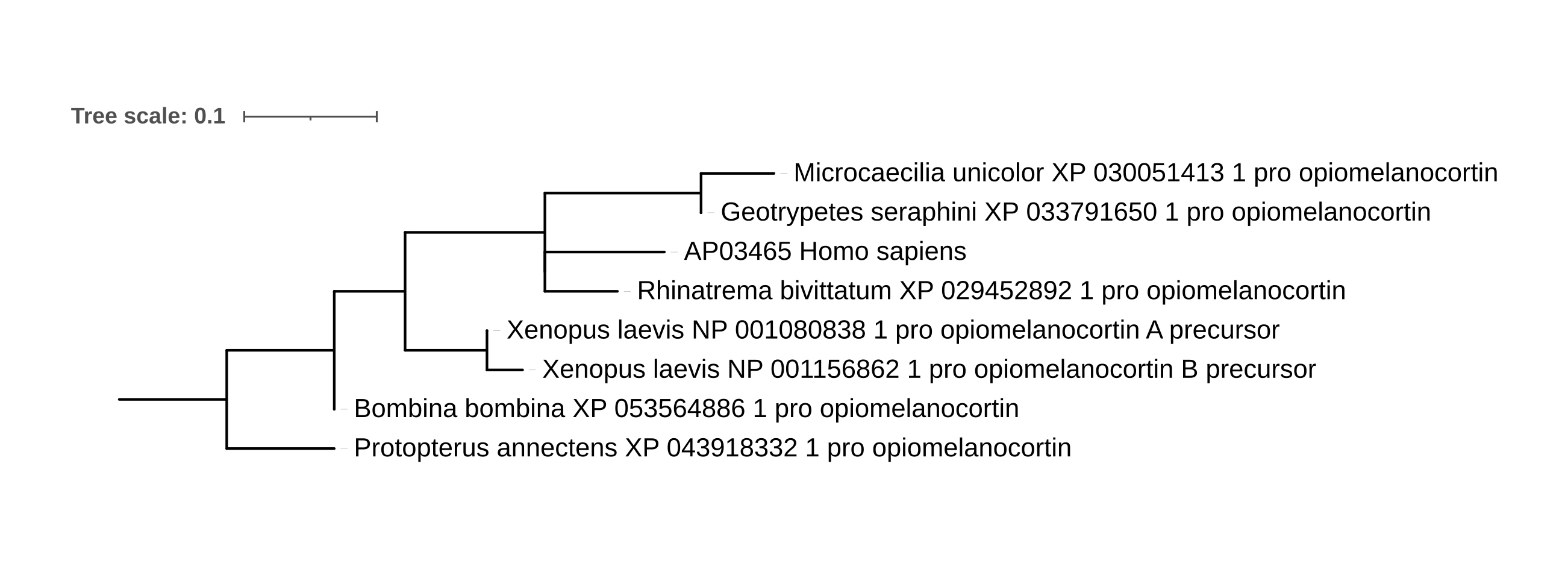

Supplement: Supplementary file 1 [file toxins-16-00150-s001.zip › Supplementary_Figure_S22.jpg]

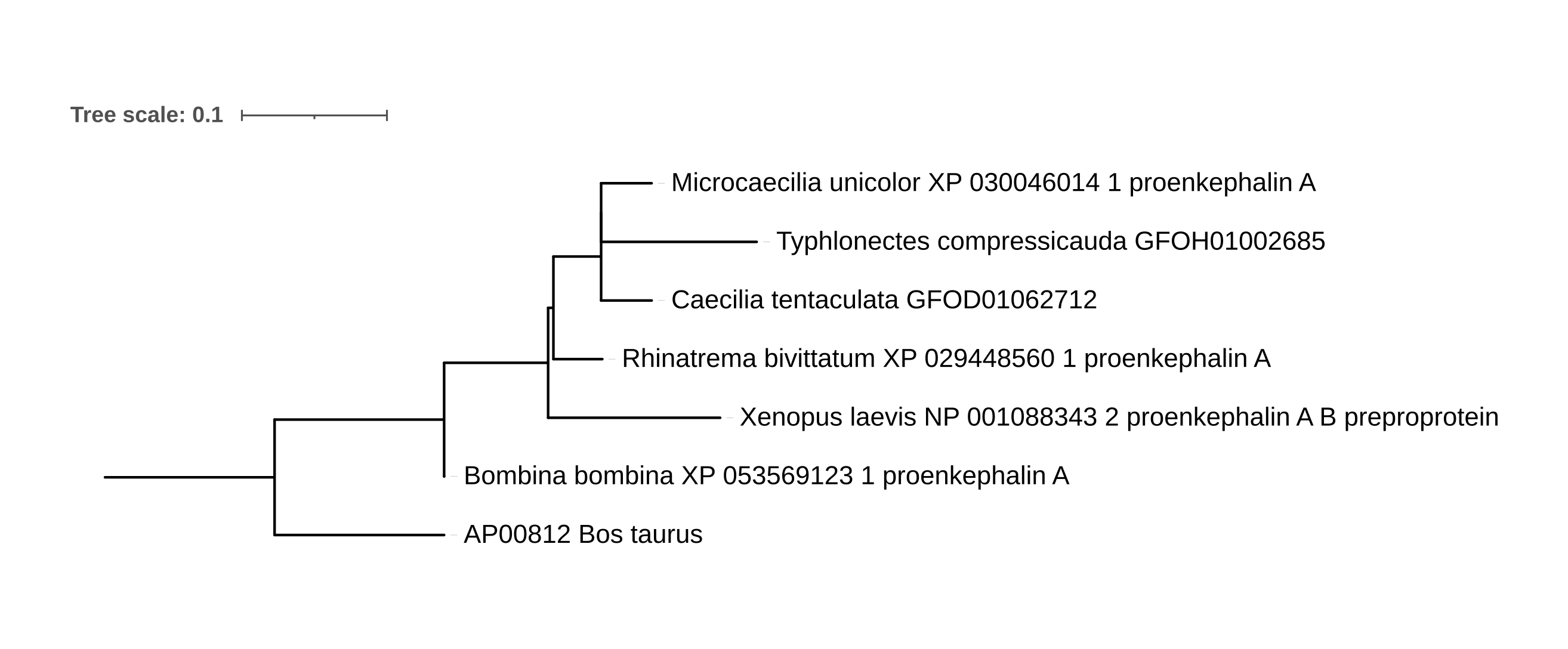

Supplement: Supplementary file 1 [file toxins-16-00150-s001.zip › Supplementary_Figure_S23.jpg]

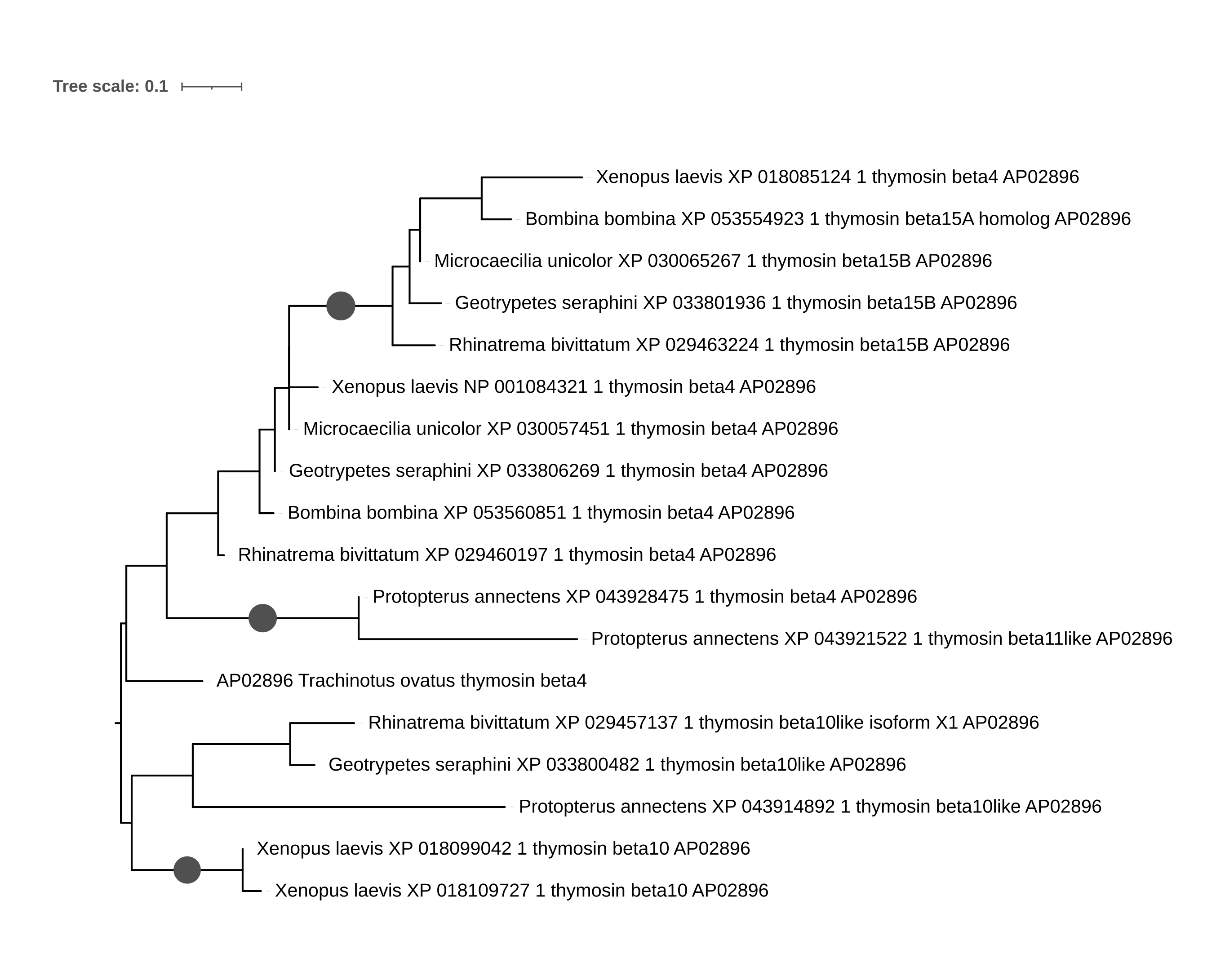

Supplement: Supplementary file 1 [file toxins-16-00150-s001.zip › Supplementary_Figure_S24.jpg]

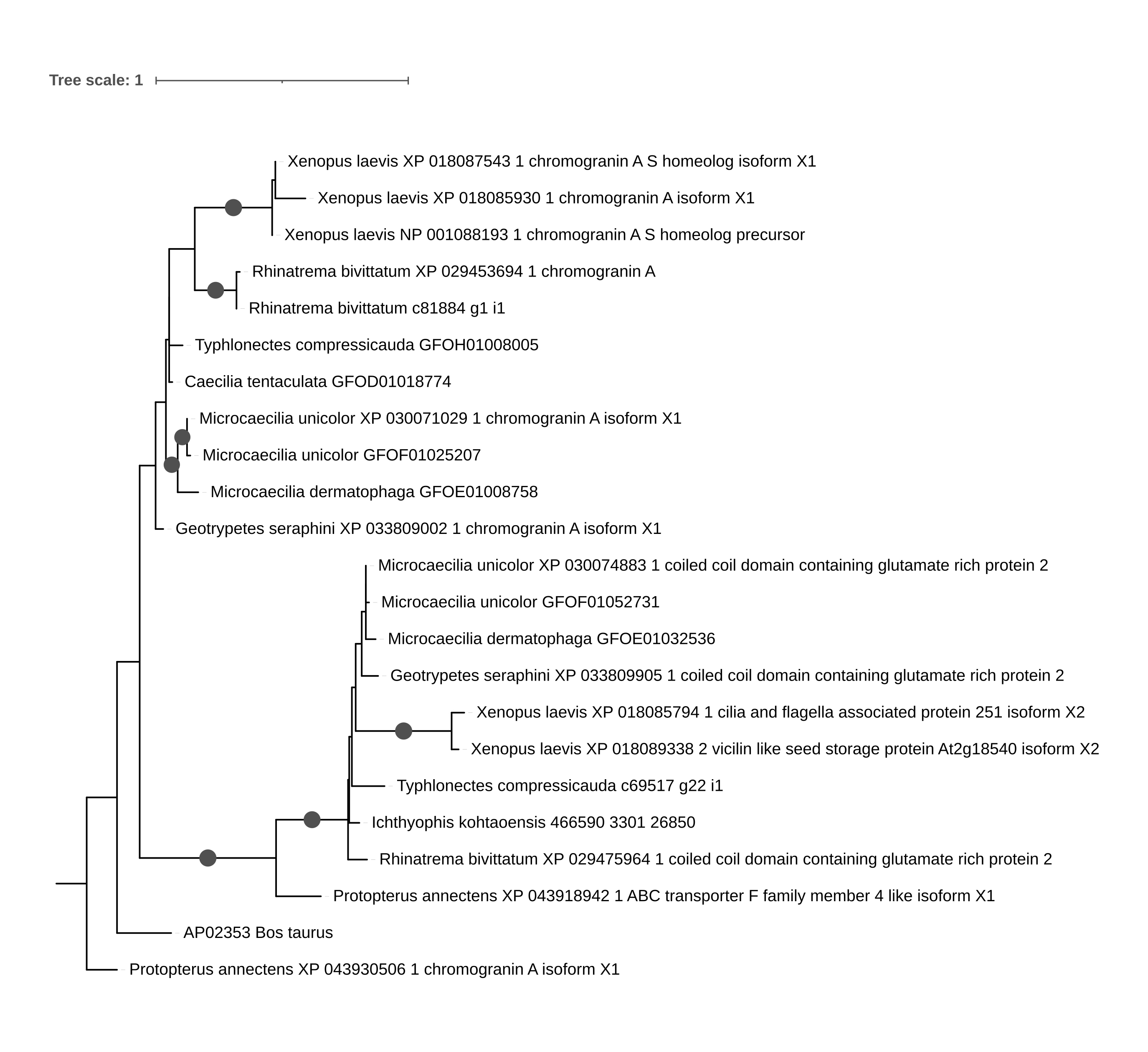

Supplement: Supplementary file 1 [file toxins-16-00150-s001.zip › Supplementary_Figure_S25.jpg]

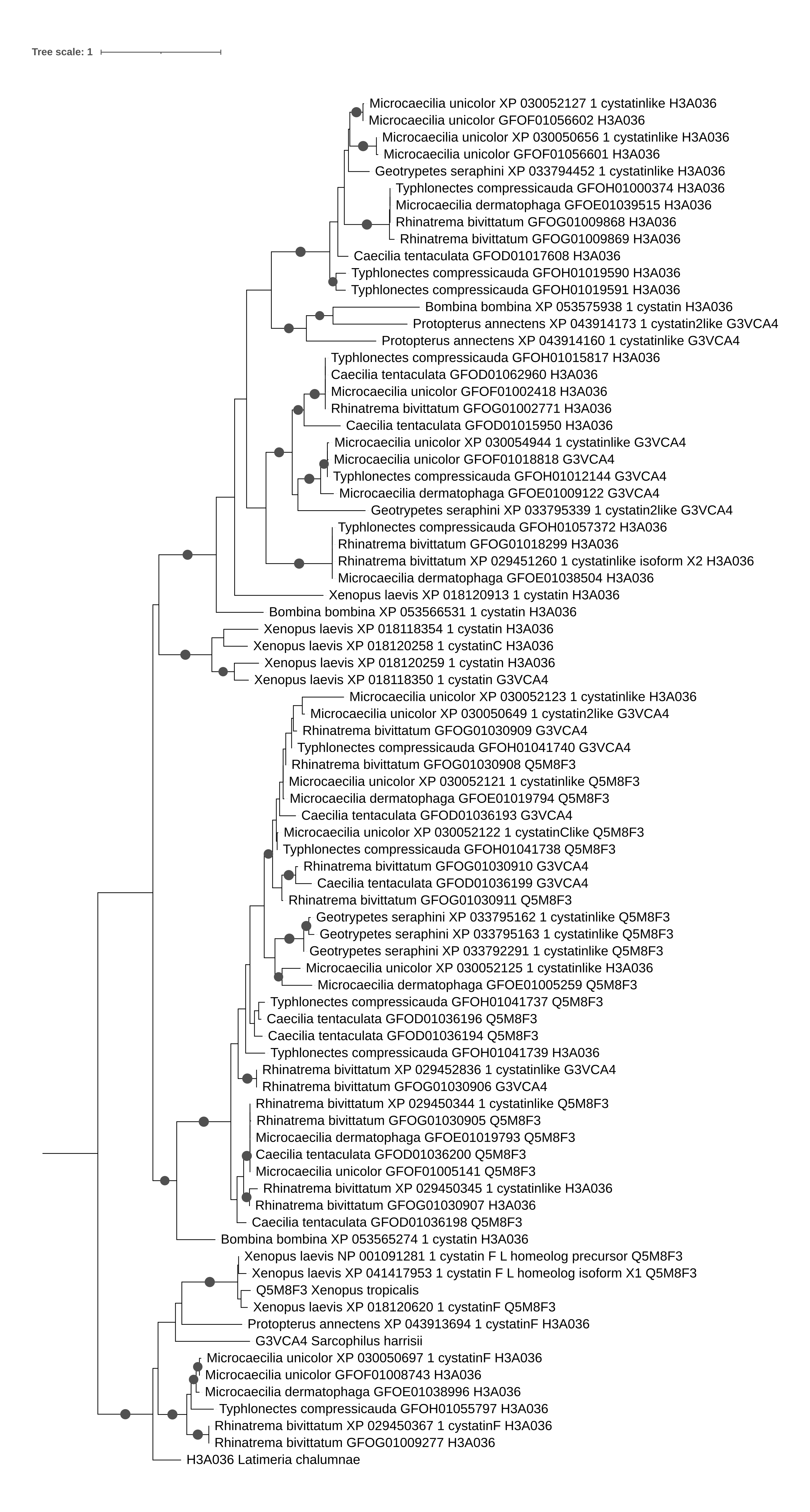

Supplement: Supplementary file 1 [file toxins-16-00150-s001.zip › Supplementary_Figure_S26.jpg]

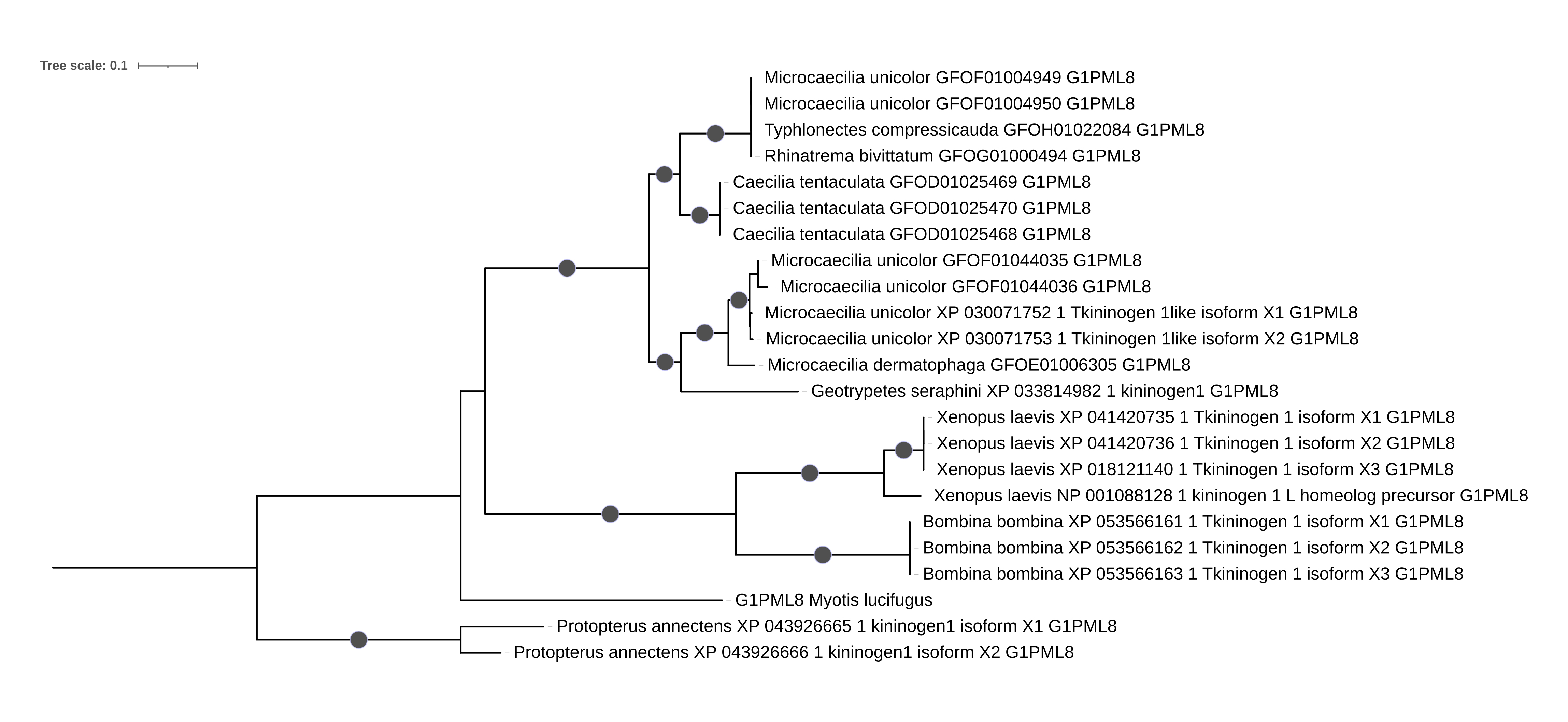

Supplement: Supplementary file 1 [file toxins-16-00150-s001.zip › Supplementary_Figure_S27.jpg]

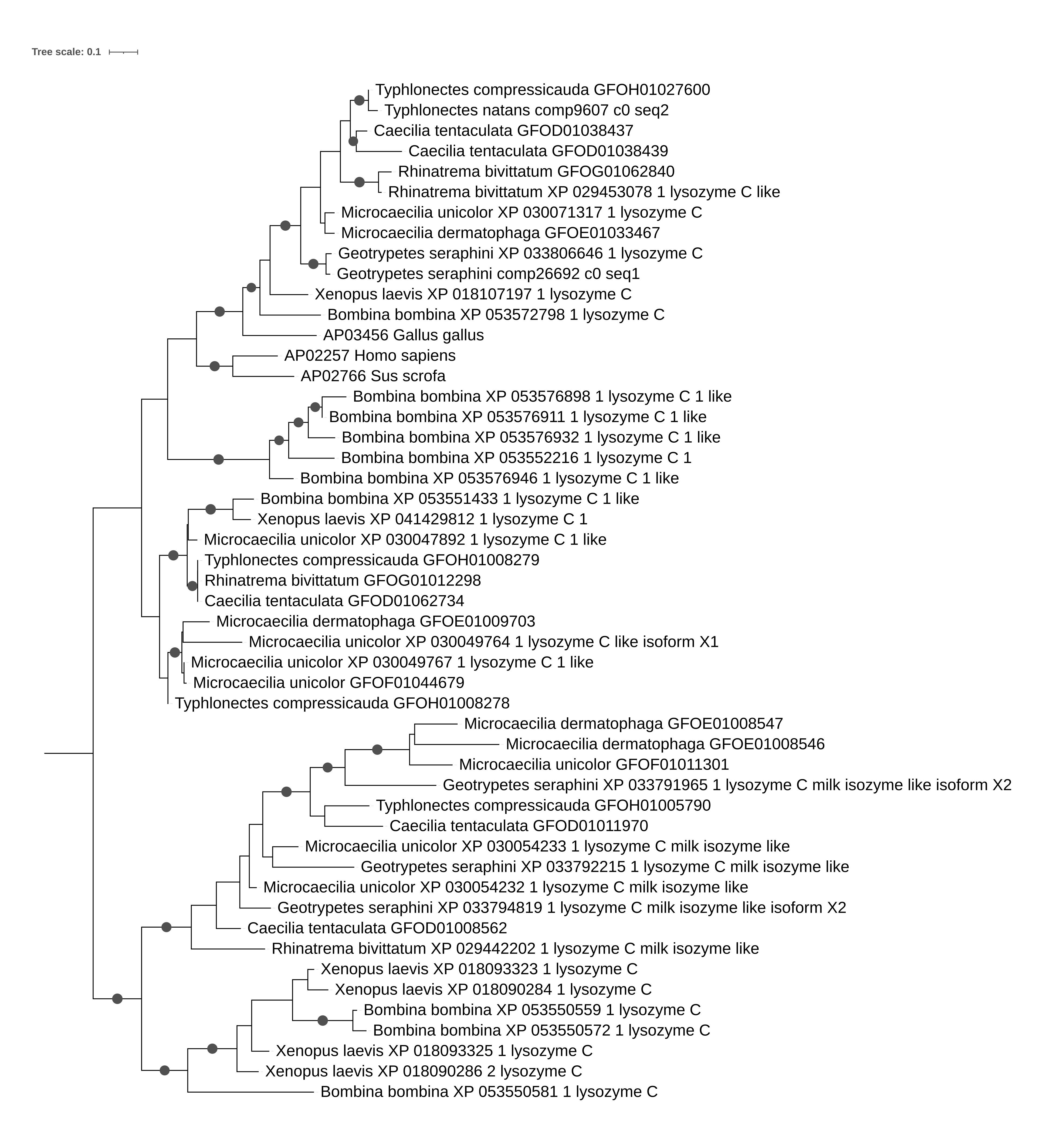

Supplement: Supplementary file 1 [file toxins-16-00150-s001.zip › Supplementary_Figure_S3.jpg]

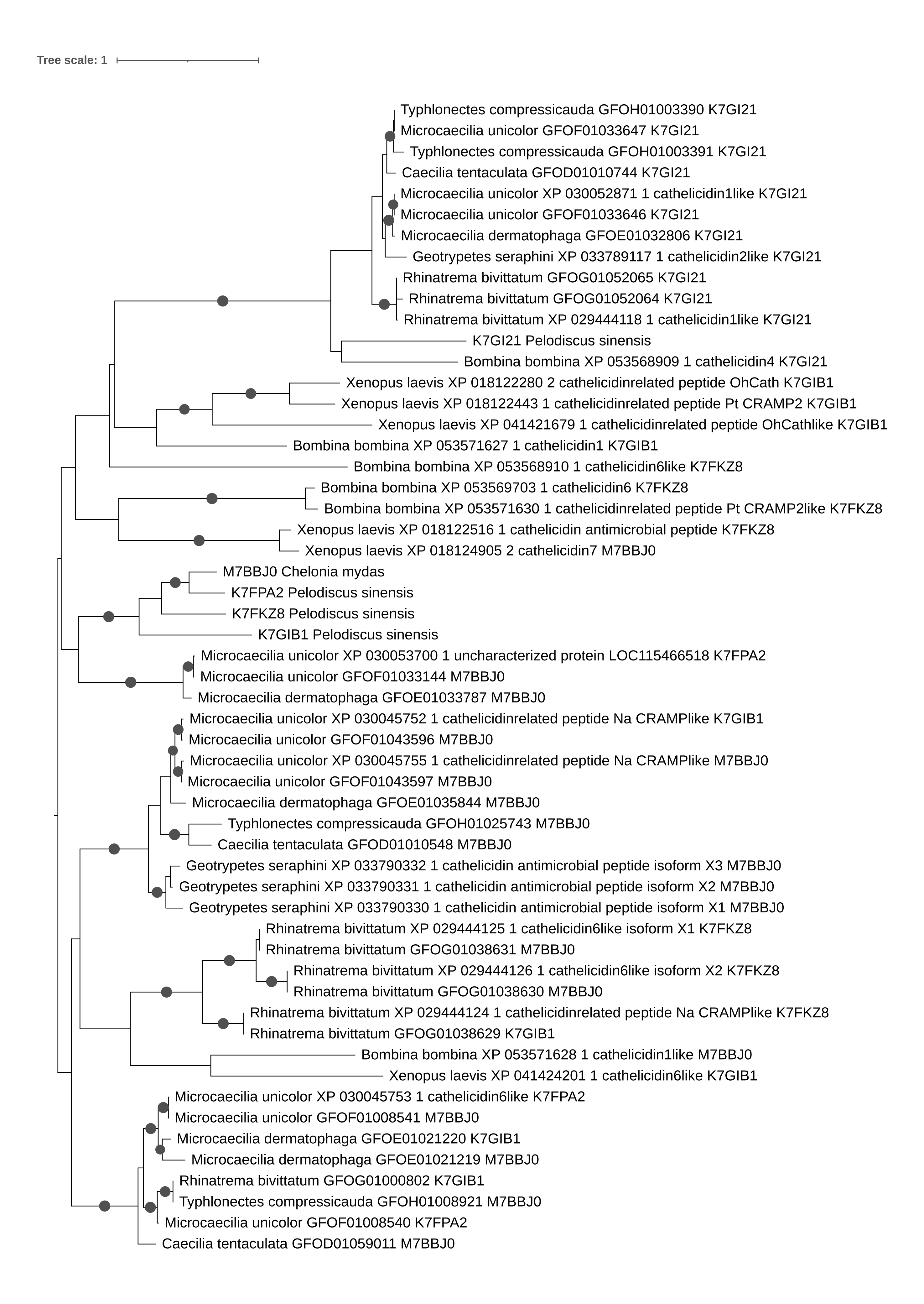

Supplement: Supplementary file 1 [file toxins-16-00150-s001.zip › Supplementary_Figure_S4.jpg]

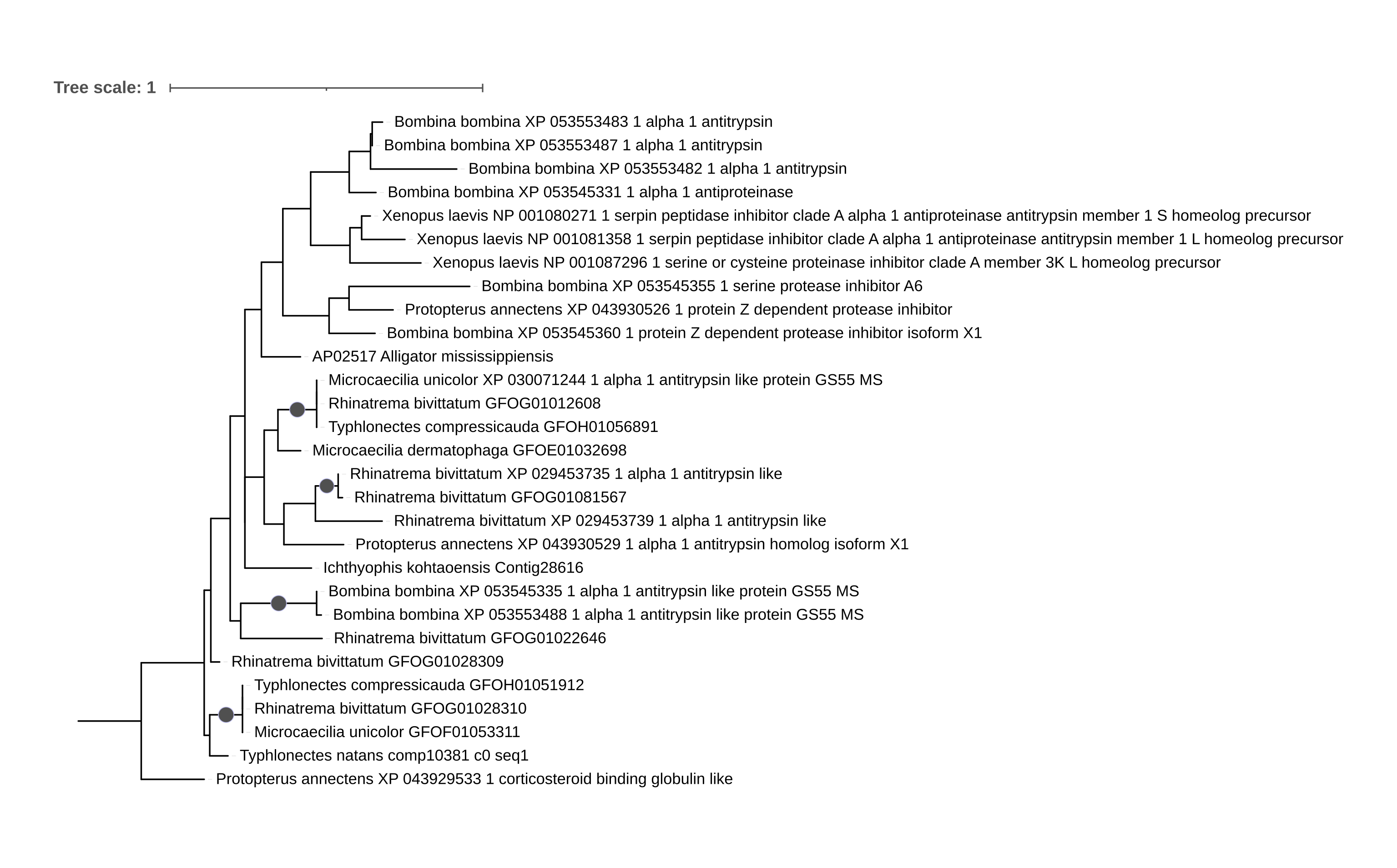

Supplement: Supplementary file 1 [file toxins-16-00150-s001.zip › Supplementary_Figure_S5.jpg]

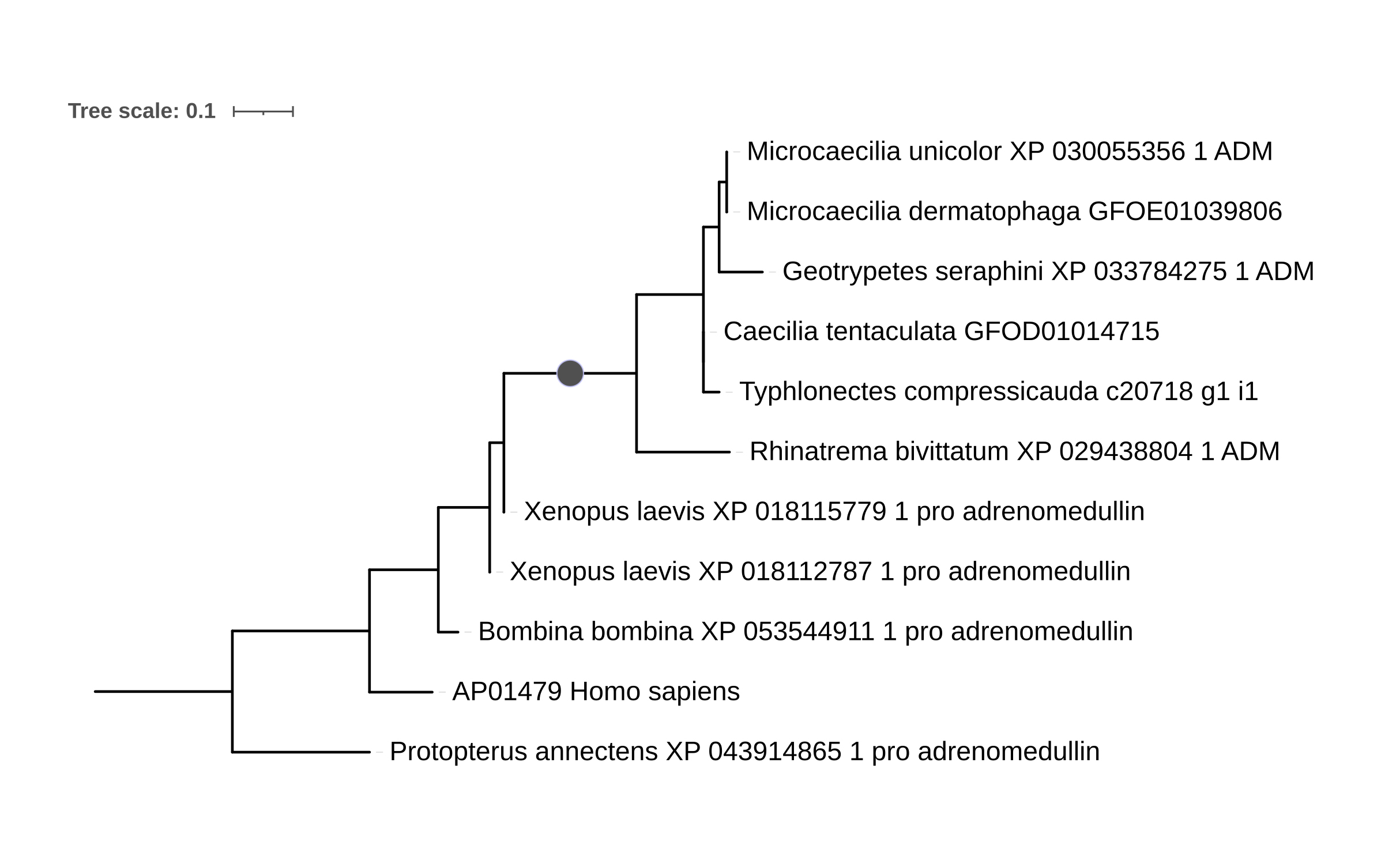

Supplement: Supplementary file 1 [file toxins-16-00150-s001.zip › Supplementary_Figure_S6.jpg]

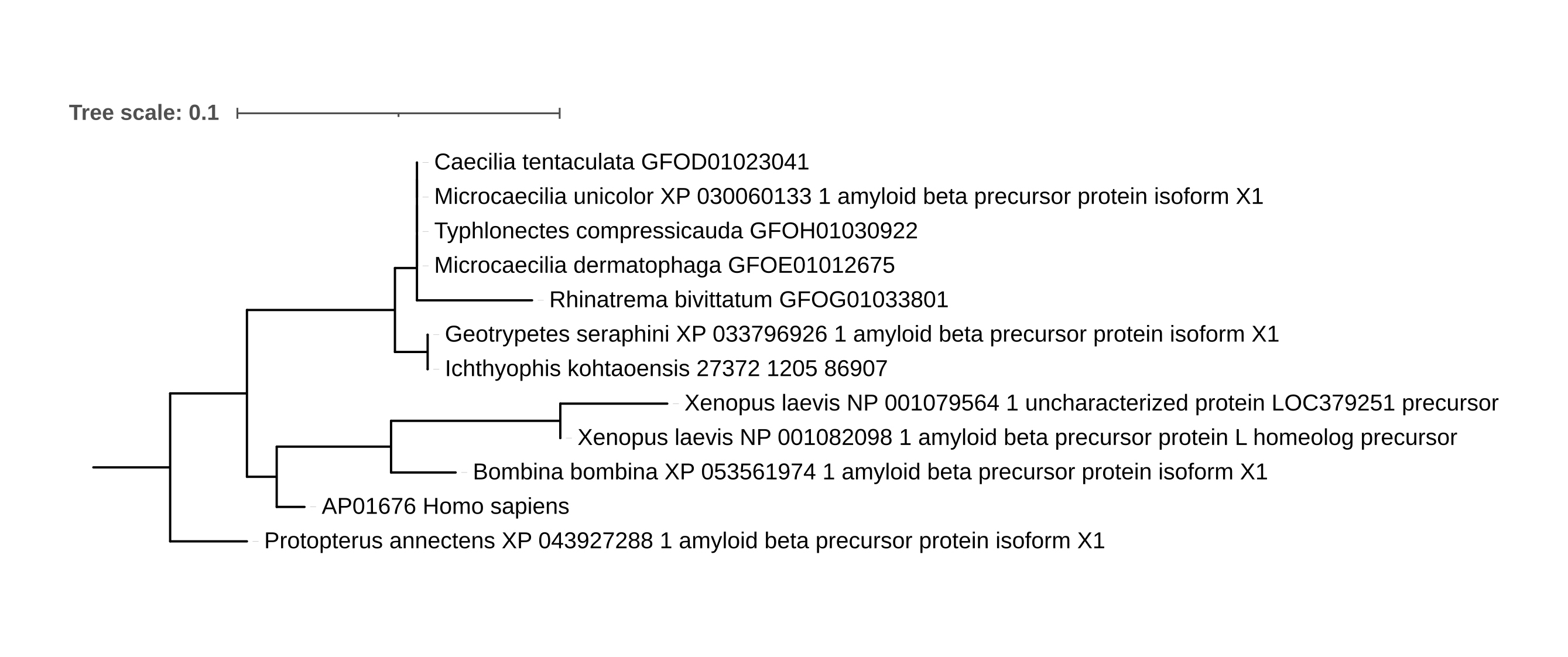

Supplement: Supplementary file 1 [file toxins-16-00150-s001.zip › Supplementary_Figure_S7.jpg]

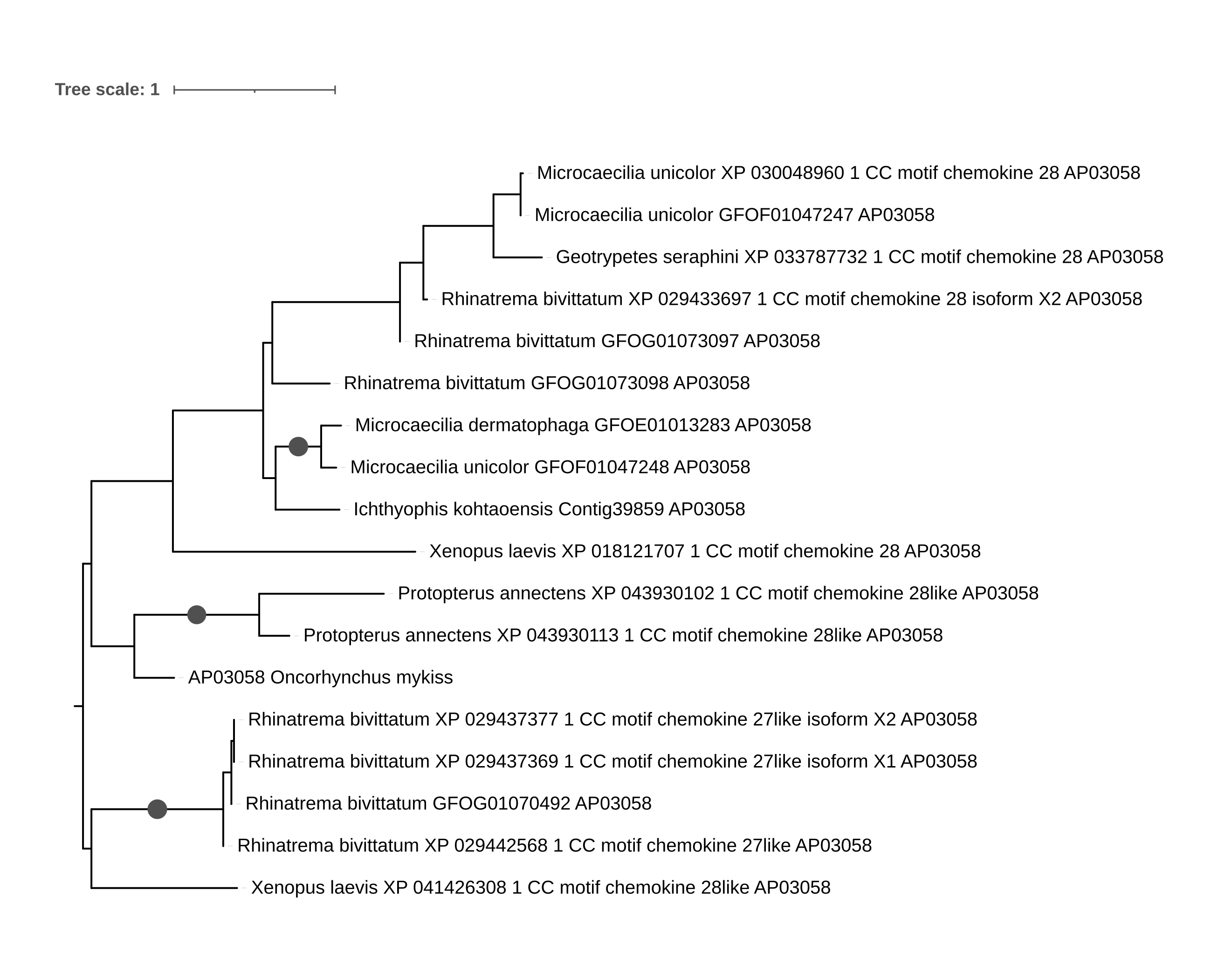

Supplement: Supplementary file 1 [file toxins-16-00150-s001.zip › Supplementary_Figure_S8.jpg]

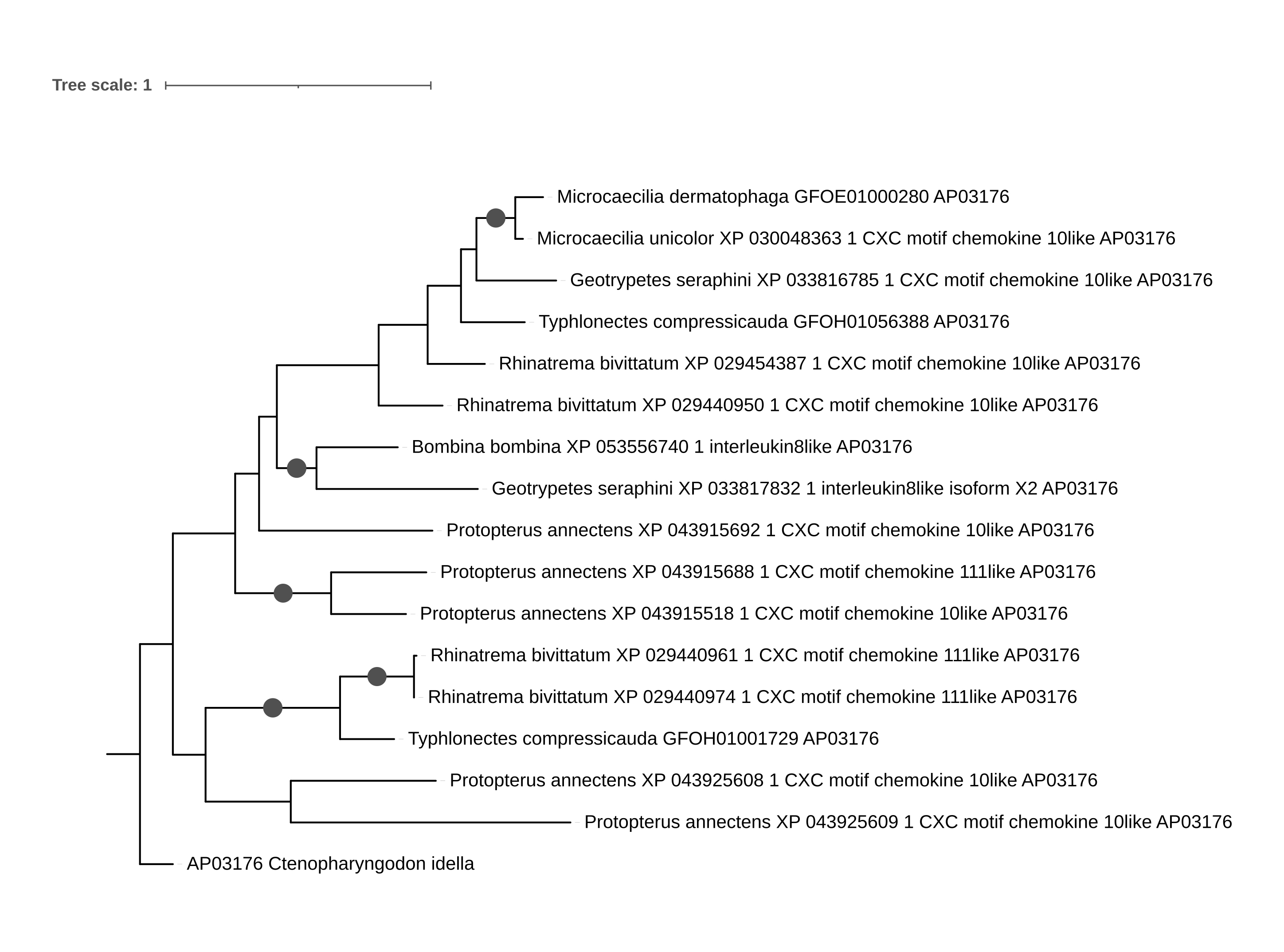

Supplement: Supplementary file 1 [file toxins-16-00150-s001.zip › Supplementary_Figure_S9.jpg]
